# The evolution of resistance to synergistic multi‐drug combinations is more complex than evolving resistance to each individual drug component

**DOI:** 10.1111/eva.13608

**Published:** 2023-11-15

**Authors:** Natalie Ann Lozano‐Huntelman, Austin Bullivant, Jonathan Chacon‐Barahona, Alondra Valencia, Nick Ida, April Zhou, Pooneh Kalhori, Gladys Bello, Carolyn Xue, Sada Boyd, Colin Kremer, Pamela J. Yeh

**Affiliations:** ^1^ Department of Ecology and Evolutionary Biology University of California, Los Angeles Los Angeles California USA; ^2^ Santa Fe Institute Santa Fe New Mexico USA

**Keywords:** antibiotic combinations, antibiotic resistance, antimicrobial, bacterial evolution

## Abstract

Multidrug antibiotic resistance is an urgent public health concern. Multiple strategies have been suggested to alleviate this problem, including the use of antibiotic combinations and cyclic therapies. We examine how adaptation to (1) combinations of drugs affects resistance to individual drugs, and to (2) individual drugs alters responses to drug combinations. To evaluate this, we evolved multiple strains of drug resistant *Staphylococcus epidermidis* in the lab. We show that evolving resistance to four highly synergistic combinations does not result in cross‐resistance to all of their components. Likewise, prior resistance to one antibiotic in a combination does not guarantee survival when exposed to the combination. We also identify four 3‐step and four 2‐step treatments that inhibit bacterial growth and confer collateral sensitivity with each step, impeding the development of multidrug resistance. This study highlights the importance of considering higher‐order drug combinations in sequential therapies and how antibiotic interactions can influence the evolutionary trajectory of bacterial populations.

## 1 INTRODUCTION

Antibiotic resistance is a problem facing humanity on a global scale (Ahmad & Khan, 2019; Coates et al., 2020; Hernando‐Amado et al., 2019; Maillard et al., 2020; Ventola, 2015). Not only is drug resistance to a single drug a threat to public health, but even more concerning, multidrug resistance in bacteria is reducing the number of viable treatment options. Some estimates show that by 2050, there will no longer be any effective antibiotic options unless new drugs are developed or discovered (Jin et al., 2020). Multiple strategies have been suggested to mitigate this ever‐growing problem, including cycling through antibiotic treatments (Nichol et al., 2015) and the use of combinational drug therapy (e.g., using multiple antibiotics simultaneously or combining antibiotics with alternative therapies, such as phage therapy) (Vivas et al., 2019). In designing new therapies, major considerations include whether or not to use two or more antibiotics at the same time, in succession, or both, as well as how much of each drug to use. We are especially interested in determining whether particular strategies (out of a world of possibilities) can reduce the emergence of antibiotic resistance. This requires investigating if and how adaptation to individual antibiotics differs from adaptation to drug combinations, and what patterns of resistance this confers on evolved populations.

Resistance of bacteria to single antibiotics has long been a topic of study. Typically, for a single antibiotic, resistance is quantified by the minimum inhibitory concentration (MIC). The MIC is when the concentration of an antibiotic that reduces growth to 0%–10% when compared to growing in an antibiotic free‐environment, depending on the specific method used when evaluating growth (Eagle & Musselman, 1948; Garrod, 1935; Haight & Finland, 1952; Thomson & Sanders, 1994). However, when multiple antibiotics with multiple mechanisms of action are used in combination, there are added complexities and nonlinearities (Beppler et al., 2016, 2017; Loewe, 1953; Tekin et al., 2016). Now, the cell is no longer facing one specific mechanism to evolve resistance to but rather multiple mechanisms, which may require multiple mutations that confer resistance.

Antibiotic resistance is acquired in two primary ways: through spontaneous mutation or through horizontal gene transfer (Blair et al., 2015). No matter how resistance evolves initially, the subsequent mutations can affect the outcomes of future antibiotic exposures (Barbosa et al., 2017; Gomez et al., 2017; Munck et al., 2014; Nichol et al., 2015, 2019; Santos‐Lopez et al., 2019). If specific mutations gained from adapting to one antibiotic confer a higher resistance to a different antibiotic, then a bacterial strain has gained cross‐resistance (Haight & Finland, 1952; Obolski et al., 2015; Sanders, 2001). If these mutations instead reduce a strain's resistance to a different antibiotic, then it is termed collateral sensitivity (Obolski et al., 2015; Pál et al., 2015). Exploiting these relationships to find combinations of antibiotics to cycle through different treatment regimen can be one potential strategy to address the antibiotic resistance problem (Brown & Nathwani, 2005; Kavanaugh et al., 2021; Kollef, 2001; Masterton, 2005; Nichol et al., 2015, 2019). This is because one goal of the cycle is always to have at least one viable antibiotic regimen available, thus locking the bacterial population in a constant state of susceptibility to current drug options.

Another proposed method for addressing multidrug antibiotic resistance is through the use of simultaneous antibiotic combinations (Vivas et al., 2019). Antibiotic combinations can be categorized by three main types of interactions resulting from their combined effect. (1) An additive combination's effects are directly predictable from combining the known, individual effects of the drugs employed. (2) A synergistic combination yields an effect that is stronger than the additive effect (Yeh et al., 2009). Synergistic combinations impose stronger selection, making it more likely that resistance mutations will sweep quickly through a population (Orr, 2000; Pepin & Wichman, 2008), and can cause faster adaptation rates (Hegreness et al., 2008). (3) Finally, an antagonistic combination yields an effect weaker than the additive effect. These antagonistic combinations require larger doses to have the same killing efficiency as synergistic combinations (Chait et al., 2007; Hegreness et al., 2008; Michel et al., 2008; Yeh et al., 2009).

The different ways in which antibiotics interact may have distinct implications for the evolution of resistance. Intriguingly, antagonistic combinations have been shown to have several advantages for slowing the evolution of resistant strains (Hegreness et al., 2008; Maillard et al., 2020; Michel et al., 2008; Ventola, 2015). Synergistic two‐drug combinations have been shown to increase the likelihood of drug resistance evolving via spontaneous mutations (Michel et al., 2008). Conversely, antagonistic combinations show a decrease in the likelihood of resistance evolving (Michel et al., 2008). Additionally, Hegreness et al. (2008) showed that antagonistic two‐drug combinations slowed the rate of resistance evolution when compared to the rate of resistance evolution to two‐drug synergistic combinations. These findings highlight how antibiotic interactions can be leveraged to slow the evolution of resistance to multidrug antibiotic combinations. Most studies have focused on two‐drug combinations, creating a knowledge gap around higher‐order combinations, that is, combinations with three or more drugs (Tekin et al., 2017; Tekin, Yeh, et al., 2018).

In multidrug combinations, many factors contribute to the overall fitness effect of the combination. The first factors to consider are the effects of each drug acting alone. Next, we must consider the effects of the additive interactions between the smaller sub‐sets of antibiotics and the other single antibiotics (or combination of antibiotics) in the mix. Finally, we need to examine the highest‐order emergent effect—that is, the effect of the interaction between all drugs present in the combination. All these factors influence how a combination will ultimately affect a bacterial population.

Numerous studies have examined the evolutionary effects of cross‐resistance and collateral sensitivity among single antibiotics in multiple species (Barbosa et al., 2017; Gomez et al., 2017; Munck et al., 2014; Nichol et al., 2015). The trajectory of resistance evolution can be influenced using knowledge of collateral effects to create a specific sequence or cycle of antibiotics to steer it away from resistance evolution (Nichol et al., 2015). In addition, some mutations, such as those affecting the ribosome, increase the evolution of multidrug resistance (Gomez et al., 2017). Yet other studies have questioned just how predictable are the collateral effects of resistance evolution. These studies have found high amounts of stochasticity and variability of these collateral effects in replicate populations (Barbosa et al., 2017; Nichol et al., 2019). Studies have also expanded into evaluating how pairwise combinations align with cross‐resistance and collateral sensitivity (Munck et al., 2014; Raymond, 2019). These studies found that bacterial populations exposed to collaterally sensitive antibiotic combinations are less likely to evolve resistance to the combination. Conversely, antibiotic pairs with similar or the same cellular/physiological targets that result in cross‐resistance tend to increase the likelihood of evolution of resistance to the combination (Munck et al., 2014).

When antibiotics are used in combinations, they are typically used at concentrations near or above the MIC (Kleine et al., 2017; Martin‐Loeches et al., 2010; Paul et al., 2014). It was previously thought that weakening the selection pressure using lower antibiotic concentrations might curb the rapid evolution of resistance. However, for some antibiotic combinations, the opposite is true. Short‐term higher doses are more effective at preventing resistance evolution compared to weaker doses (Bollenbach, 2015). When exposed to one antibiotic, at sub‐inhibitory concentrations, an SOS response system is induced in bacteria. This SOS response allows for damaged DNA to be bypassed by the cell which in turn allows for mutation rates to increase (Chow et al., 2021). Thus, the effect of combinations could also be dependent on the dosages.

Our study focuses on *Staphylococcus epidermidis*, a gram‐positive bacterium that colonizes skin and mucosa. *S. epidermidis* was previously considered an innocuous commensal microorganism on the human skin. However, it has recently become an opportunistic pathogen that results in nosocomial infections, particularly in indwelling medical devices such as catheters (Otto, 2009). We investigate the effects of resistance evolution to a single antibiotic and combinations of antibiotic used simultaneously. We examine four highly synergistic three‐drug combinations where all antibiotics are at sub‐inhibitory concentrations (<30% inhibition). These combinations are made up of piperacillin and tetracycline, with a third antibiotic of either: chloramphenicol, doxycycline, erythromycin, or neomycin. Specifically, we address the following questions: (1) How does evolved resistance to a three‐drug combination affect subsequent sensitivity to individual components of the combination? (2) Conversely, how does evolved resistance to individual components of a three‐drug combination affect sensitivity to the combination? (3) How can the interactions within a three‐drug combination change after bacteria evolve resistance to one part of the antibiotic combination?

## 2 MATERIALS AND METHODS

### 2.1 Creation and isolation of resistant mutants

Eight strains of resistant *S. epidermidis* (ATCC 14990) were independently evolved in a stepwise manner to each of six antibiotics (Table 1) and each of four focal three‐drug combinations known to be highly synergistic (Table 2). From here onward, individual antibiotics will be spelled out while combinations of antibiotics will be listed using their abbreviations (Table 1). For example, a combination consisting of piperacillin, tetracycline, and erythromycin will be listed as PIP + TET + ERY. We use the term drug regimen to be a single antibiotic or combination of antibiotics used simultaneously to inhibit bacterial growth and the term treatment to encompass a set of drug regimens (in sequential order or not) that can decrease bacterial growth.

**TABLE 1:** Antibiotic list and properties on the ancestral strain of *Staphylococcus epidermidis* (ATCC 14990).

Antibiotic (abbreviation)	Class	Mechanism of action	Concentration used (μM)	Relative fitness to No drug control	MIC (μM)
Chloramphenicol (CHL)	Chloramphenicol	Protein Synthesis, 50S	90	0.989	1352.621
Doxycycline (DOX)	Tetracycline	Protein Synthesis, 30S	0.6	0.780	257.522
Erythromycin (ERY)	Macrolide	Protein Synthesis, 50S	0.05	1.000	0.511
Neomycin (NEO)	Aminoglycoside	Protein Synthesis, 30S	0.35	0.803	13.280
Piperacillin (PIP)	β‐Lactam	β‐Lactam, Cell wall	0.6	0.966	1.627
Tetracycline (TET)	Tetracycline	Protein Synthesis, 30S	20	0.713	205.137

**TABLE 2:** Drug concentrations of synergistic three‐drug combinations with net interaction (*DA*) and emergent interaction (*E*_3_) values are based on the ancestral strain of *Staphylococcus epidermidis* (ATCC 14990).

Three‐drug combination	Drugs in combination (abbreviation)	Single drug concentration (μM)	Fitness effect of the combination	Net interaction (*DA*)	Emergent interaction (*E*_3_)
1	(A) Piperacillin (PIP)	0.6	0.004	−0.97	−0.09
	(B) Tetracycline (TET)	20			
	(C) Chloramphenicol (CHL)	90			
2	(A) Piperacillin (PIP)	0.6	0	−1	1
	(B) Tetracycline (TET)	20			
	(C) Doxycycline (DOX)	0.6			
3	(A) Piperacillin (PIP)	0.6	0	−1	0.98
	(B) Tetracycline (TET)	20			
	(C) Erythromycin (ERY)	0.05			
4	(A) Piperacillin (PIP)	0.6	0	−1	−0.08
	(B) Tetracycline (TET)	20			
	(C) Neomycin (NEO)	0.35			

To start the initial populations, we prepared a highly dense cell culture of *S. epidermidis* by pinning the parental strain of *S. epidermidis* into 200 μL per well of a 96‐well plate of Lysogeny Broth (LB) media (10 g tryptone, 5 g yeast extract, and 10 g NaCl) and incubated the culture for ~16 h at 37°C shaking at 130 rpm. To evolve resistance to a single antibiotic (those listed in Table 1), the highly dense cell culture was then pinned over to a 96‐well plate containing 200 μL per well of LB and the antibiotic concentration for day 1, beginning with 50% of the parental minimum inhibitory concentration (MIC). The antibiotic concentration was continually doubled every 48 h over 10 days, which is roughly 100 generations, resulting in a final drug concentration of 800% of the parental MIC. To evolve combination ‐resistant strains, we also began with the parental strain of *S. epidermidis*. Similar to the creation of our single‐drug‐resistant mutants, we utilized a high‐density cell culture of the parental strain. These high‐density cell cultures were then pin‐transferred into wells of a new 96‐well plate filled with 200 μL of LB media with one of the four, three‐drug combinations, as listed in Table 2. These 96‐well plates were then incubated at 37°C for approximately 24 h. Pinning the cell cultures into fresh media and antibiotics occurred every 24 h and continued for 10 days or approximately 100 generations. Drug concentrations for all different drug combinations remained at constant levels for the entire time of the evolution experiment. This was done to keep the interactions within a combination as constant as possible allowing only for adaptations in the populations to influence how an interaction may change (Berenbaum et al., 1983).

Once the 10‐days experimental evolution component concluded, we isolated a single colony from each independently evolved population (each well). For the antibiotic chloramphenicol, resistant mutants were collected at 400% of the parental MIC, since growth would not occur at any higher concentrations. We isolated and confirmed resistance by streak purifying onto LB agar with 800% parental MIC of the respective antibiotic (400% parental MIC for chloramphenicol) or the respective antibiotic combination. We took the selected colonies from the streak purification, grew them in 2 mL Luria Broth and incubated the culture for ~16 h at 37°C shaking at 160 rpm. This cell culture was then re‐purified again on LB agar with the respective 800% (or 400% for CHL) parental MIC of the respective drug or the respective antibiotic combination. Master tubes with aliquots were made from a single colony selected from each of the second purifications that were cultured and stored at −80°C (in 25% glycerol).

### 2.2 Determination of the dose–response curve

**FIGURE 1:**
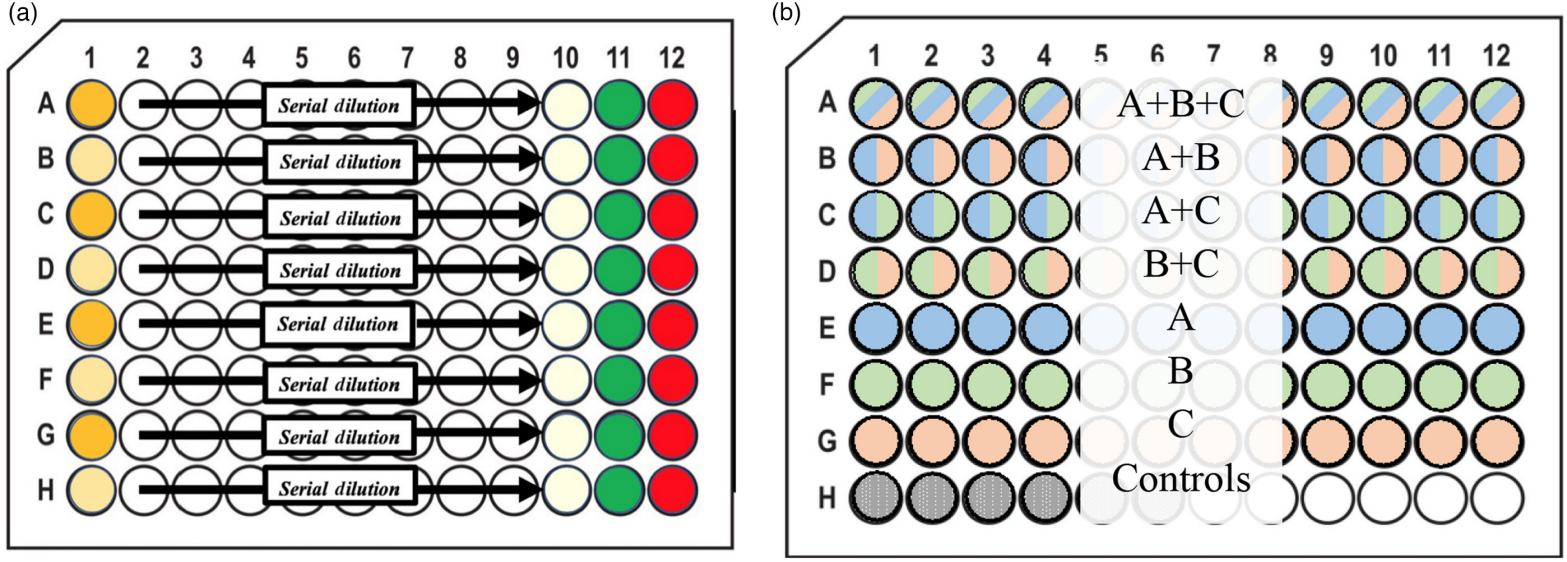
Plate Layouts for the MIC estimates and Deep Well Plates. (a) This figure shows each drug in concentration value in the 96‐well plates during the trials for evaluating the MIC of each bacterial strain. (b) The diagram above illustrates an example of the layout used—a single deep well plate—during the trials. This figure includes the locations for the high‐ and lower‐order drug combinations and their controls all of which are required to determine emergent interactions. These deep well plates were incubated for 22 h at 37°C and later transferred onto a flat bottom 96‐well plates for OD reading.

The dose–response curves were determined using a 20‐step, two‐fold serial dilution of the antibiotics starting at 2000 μg/mL. The layout for determining the dose–response curves of all bacterial strains is seen below in Figure 1a. Each well had a total working volume of 200 μL, was inoculated with 50 μL 1 × 10^5^ CFU/mL, and incubated at 37°C for 22 h (Reller et al., 2009). A total of four technical replicates for each dose–response curve determination were done. The data from all four replicates were pooled together into the “drc” package in R to model the dose–response curve (Ritz et al., 2015, 2016). This model was then used to estimate the MIC and the concentration of antibiotics needed to inhibit growth by 50% (IC_50_). We also included negative controls on each of the 96‐well plates to confirm that there was no contamination of our media.

### 2.3 Combination interaction determination

The single‐drug resistant mutants showed a phenotype that aggregates at the bottom of the standard flat well plates. This is not uncommon when resistance evolves (Cushnie et al., 2007; Dastgheyb et al., 2015; Haaber et al., 2012; Lozano‐Huntelman et al., 2019; Ritz et al., 2015; Secor et al., 2018). This aggregation did not allow for an accurate optical density, OD, reading that correlates to the number of cells in the culture. To obtain accurate OD readings, we used deep 96‐well plates for the drug combinations so we could resuspend the cells in the media without creating bubbles. The plate set‐up includes all higher‐ and lower‐order combinations along with positive and negative controls as a means to compare the relative growth of bacteria (Figure 1b) which is required for the framework used to determine the interaction values (see next paragraph for more information on this framework). The total working volume of these deep well plates was 400 μL and each well was comprised of 100 μL of LB and 100 μL of the drug combination at their specified concentration, which is described in Table 2. Additionally, these deep‐well plates were inoculated with 200 μL 1 × 10^5^ CFU/mL and incubated for 24 h at 37°C. After the 24‐h incubation period was complete, 200 μL of the culture was transferred to a flat bottom 96‐well plate to gather OD readings.

To calculate net and emergent interactions, we followed the Rescaled Bliss Independence (RBI) framework outlined in Beppler et al. (2016) and Tekin et al. (2016). Other approaches have also been used, such as Loewe Additivity (Loewe, 1953), which, unlike RBI, assumes that a drug cannot interact with itself. However, the RBI framework was chosen for its unique ability to determine the emergent *E*_3_ value (described below) that quantifies how much of the interaction is emerging from having all three drugs in combination, rather than from a strong interaction from a two‐drug combination (Beppler et al., 2016; Tekin et al., 2016). Briefly, in the RBI framework the net deviation from additivity, *DA*, is determined by only removing the fitness effects contributed by each drug alone ($w_{X},w_{Y},w_{Z}$) from the overall fitness effect ($w_{\mathrm{XYZ}}$) assuming Bliss independence (Equation 1) (Bliss, 1939). Once the net *DA* is determined, the process can be done again, removing not only the additive contributions of each drug but also the effects of all lower‐order interactions, leaving only the emergent effect (Equation 2) (Beppler et al., 2016).

(1) $$\mathrm{DA}=w_{\mathrm{XYZ}}-w_{X}w_{Y}w_{Z}$$

(2) $$E_{3}=w_{\mathrm{XYZ}}-w_{X}w_{\mathrm{YZ}}-w_{Y}w_{\mathrm{ZX}}-w_{Z}w_{\mathrm{YZ}}+2w_{X}w_{Y}w_{Z}$$

After the initial interaction value is determined, a rescaling process is used to better distinguish between interaction types (Tekin et al., 2016). The interaction values were rescaled following the same framework and methodology as used in Tekin, White, et al. (2018).

### 2.4 Whole genome sequencing and quality control

To ensure strain purity, each strain was grown overnight in the antibiotic regimens they evolved resistance to (either a single drug or in a corresponding combination). DNA extractions were performed using the “Quick‐DNA™ Fungal/Bacterial Miniprep Kit” from Zymo Research (Catalog number: D6005). We followed the manufacturer's protocols which included adding beta‐mercaptoethanol to the genomic lysis buffer. Once DNA was extracted, samples were sent to Novogene for paired‐end sequencing using Illumina's NovaSeq PE150 and paired‐end read quality was evaluated using FastQC (v0.11.9). Additionally, all paired‐end samples were subjected to metagenomic taxonomic classification using the Kraken2 algorithm (Wood et al., 2019). Samples with extensive (>5%) unclassified reads were removed from further analysis. Under this criterion, PIP1, DOX1, DOX6, and DOX7 samples were discarded from downstream analyses.

### 2.5 Variant analysis

Sample‐specific paired‐end read sets were aligned against the ancestral strain using the Burrows‐Wheeler Alignment BWA‐MEM algorithm and single or multiple nucleotide polymorphisms (SNP/MNP) mutations were detected across mutant genomes using the FreeBayes SNP caller v1.3.2‐1 (Garrison & Marth, 2012). To boost confidence in detected mutations, all gene variants with a Phred score beneath 20—corresponding to a 99% accuracy threshold—were removed from further analysis. Additionally, given the interest in identifying mutations that directly impact known protein function in evolved resistant strains, synonymous mutations and mutations residing in hypothetical gene products were excluded from downstream analysis.

### 2.6 Statistical analysis

We assessed how the evolution of resistance can change both combination susceptibility and individual antibiotic sensitivity. We measured relative growth (Equation 3) as the growth of a population exposed to an antibiotic(s) normalized to the growth of the same population grown in a drug‐free environment.

(3) $$\text{Relative growth}=\frac{\mathrm{OD}600\;\text{of treated population}}{\mathrm{OD}600\;\text{of}\;a\;\text{population in}\;a\;\mathrm{no}‐\text{drug environment}}$$

When exposing bacterial populations to the combinations, if relative growth was at or below 5%, we considered the strain to be susceptible to the combination. These definitions of relative growth and susceptibility to a combination are used consistently throughout the remainder of the paper.

To assess how the evolution of resistance to a combination (PIP + TET + CHL, PIP + TET + DOX, PIP + TET + ERY, or PIP + TET + NEO) can change individual antibiotic sensitivity, we evaluated the inhibitory concentration at 50% inhibition (IC_50_). We evaluated the fold change in IC_50_ (IC_50_ of resistant strain/IC_50_ of ansestrial strain) by performing a two‐tailed, one‐sample *t*‐test defining H_0_ as *μ* = 1. We then determined statistical significance through a Holm–Bonferroni correction. To assess how the evolution of resistance to a single drug can change the susceptibility to a combination (PIP + TET + CHL, PIP + TET + DOX, PIP + TET + ERY, or PIP + TET + NEO), we evaluated if resistance to a single drug resulted in more than 5% relative growth by performing a two‐tailed, one‐sample *t*‐tests to (using H_0_: *μ* = 0.05). We then adjusted for multiple comparisons via a Holm–Bonferroni correction.

We also examined how the interaction values may have changed when resistance to a single antibiotic evolved. We took the difference between the interaction value of an evolved strain and the interaction value of the ancestral strain, these values were then compared to zero via a two‐tailed, one‐sample *t*‐test (H_0_: *μ* = 0).

## 3 RESULTS

We cultivated eight independently evolved resistant strains of *S. epidermidis* for each individual antibiotic (tetracycline, piperacillin, chloramphenicol, doxycycline, erythromycin, or neomycin). We also created eight independently evolved resistant strains for each of the four 3‐drug combinations: PIP + TET + CHL, PIP + TET + DOX, PIP + TET + ERY, and PIP + TET + NEO. These combinations are all highly synergistic and show at least 95% inhibition of the ancestral strain (ATCC 14990).

### 3.1 Resistance to combinations leads to changes in single antibiotic susceptibility

**FIGURE 2:**
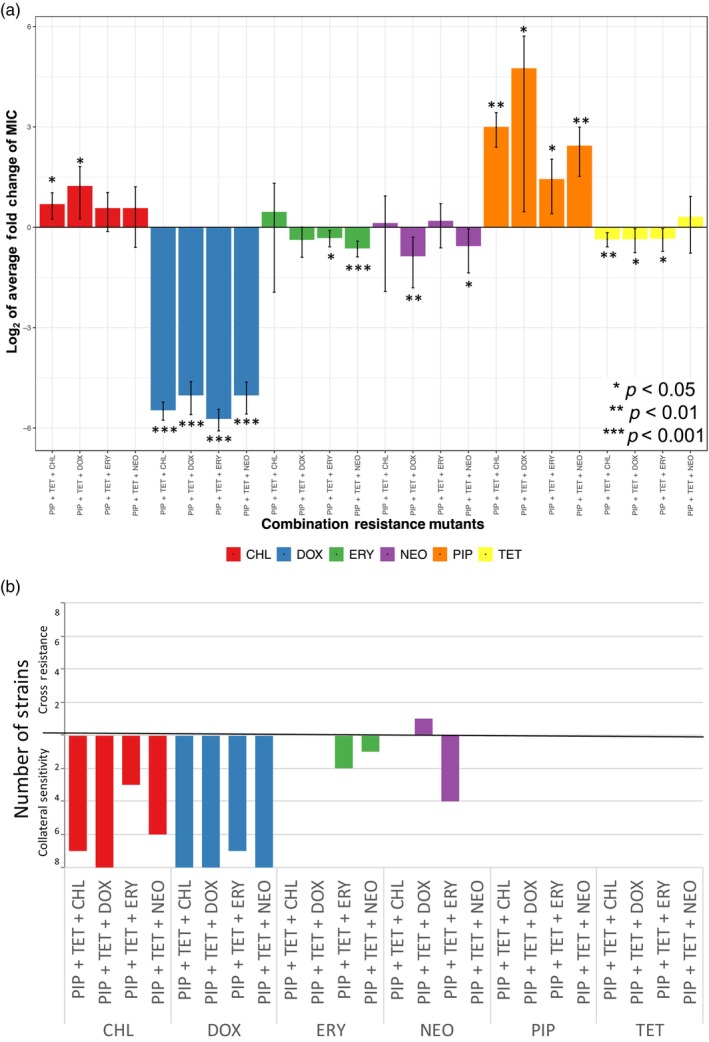
Resistance to synergistic drug combinations results in more incidences of collateral sensitivity than cross‐resistance. (a) The fold change in MIC values (MIC_resistant_/MIC_ancestral_) when strains resistance to synergistic drug combinations are exposed to a single antibiotic. (b) The number of strains that had a change in susceptibility to a single antibiotic with a *p*‐value < 0.002. This indicates a significant change in the MIC after correcting for multiple comparisons. Strains that showed cross‐resistance are above the 0 line while collaterally sensitive strains are below the 0 line.

We first examined the collateral effects of how the evolution of resistance to the combinations can influence the IC_50_ of all single antibiotics tested in this study. Over 80% of the observed changes in susceptibility (71 of 86) reflected increases in susceptibility rather than resistance (Figure 2). Further examination of strain‐specific fold changes also yielded more instances of increased sensitivity (Table 3).

**TABLE 3:** Strain‐specific relative fold change of concentration that inhibits 50% growth with the corresponding *p*‐value.

Independently evolved strain	CHL	DOX	ERY	NEO	PIP	TET
PIP + TET + CHL						
1	Fold change: 0.383	Fold change: 0.087	Fold change: 1.096	Fold change: 1.746	Fold change: 0.548	Fold change: 3.34
	*p* = 0	*p* = 0	*p* = 0.565	*p* = 0.074	*p* = 0.821	*p* = 0.024
2	Fold change: 0.251	Fold change: 0.043	Fold change: 1.360	Fold change: 2.249	Fold change: 0.581	Fold change: 1.564
	*p* = 0	*p* = 0	*p* = 0.127	*p* = 0.012	*p* = 0.883	*p* = 0.203
3	Fold change: 0.324	Fold change: 0.080	Fold change: 2.740	Fold change: 2.144	Fold change: 0.416	Fold change: 1.604
	*p* = 0	*p* = 0	*p* = 0.009	*p* = 0.016	*p* = 0.698	*p* = 0.186
4	Fold change: 0.280	Fold change: 0.063	Fold change: 1.720	Fold change: 1.562	Fold change: 0.289	Fold change: 1.320
	*p* = 0	*p* = 0	*p* = 0.035	*p* = 0.249	*p* = 0.468	*p* = 0.279
5	Fold change: 0.319	Fold change: 0.052	Fold change: 2.216	Fold change: 1.728	Fold change: 0.470	Fold change: 2.235
	*p* = 0	*p* = 0	*p* = 0.027	*p* = 0.03	*p* = 0.732	*p* = 0.055
6	Fold change: 0.548	Fold change: 0.088	Fold change: 0.995	Fold change: 1.522	Fold change: 0.492	Fold change: 1.257
	*p* = 0	*p* = 0	*p* = 0.973	*p* = 0.071	*p* = 0.752	*p* = 0.362
7	Fold change: 0.245	Fold change: 0.108	Fold change: 1.495	Fold change: 1.615	Fold change: 0.385	Fold change: 1.425
	*p* = 0	*p* = 0	*p* = 0.029	*p* = 0.102	*p* = 0.567	*p* = 0.207
8	Fold change: 0.279	Fold change: 0.061	Fold change: 0.977	Fold change: 1.330	Fold change: 0.218	Fold change: 1.185
	*p* = NA	*p* = 0	*p* = 0.885	*p* = 0.391	*p* = 0.011	*p* = 0.486
PIP + TET + DOX						
1	Fold change: 0.245	Fold change: 0.124	Fold change: 0.935	Fold change: 5.379	Fold change: 10.379	Fold change: 2.759
	*p* = 0	*p* = 0	*p* = 0.646	*p* = 0.001	*p* = NA	*p* = 0.012
2	Fold change: 0.270	Fold change: 0.102	Fold change: 1.019	Fold change: 1.712	Fold change: 0.466	Fold change: 1.292
	*p* = 0	*p* = 0	*p* = 0.905	*p* = 0.101	*p* = 0.856	*p* = 0.333
3	Fold change: 0.268	Fold change: 0.057	Fold change: 1.126	Fold change: 1.583	Fold change 0.263	Fold change: 1.178
	*p* = 0	*p* = 0	*p* = 0.565	*p* = 0.046	*p* = NA	*p* = 0.502
4	Fold change: 0.280	Fold change: 0.210	Fold change 0.894	Fold change: 1.513	Fold change: 0.226	Fold change: 1.386
	*p* = 0	*p* = 0	*p* = 0.402	*p* = 0.065	*p* = 0.141	*p* = 0.277
5	Fold change: 5.050	Fold change: 0.084	Fold change: 0.727	Fold change: 2.140	Fold change: 0.438	Fold change: 1.100
	*p* = 0.377	*p* = 0	*p* = 0.008	*p* = 0.058	*p* = 0.748	*p* = 0.97
6	Fold change: 0.269	Fold change: 0.089	Fold change: 0.847	Fold change: 3.309	Fold change: 0.326	Fold change: 1.171
	*p* = 0	*p* = 0	*p* = 0.256	*p* = 0.01	*p* = 0.649	*p* = 0.543
7	Fold change: 0.810	Fold change: 0.087	Fold change: 0.835	Fold change: 1.373	Fold change: 0.244	Fold change: 2.065
	*p* = 0.002	*p* = 0	*p* = 0.176	*p* = 0.104	*p* = 0.065	*p* = 0.072
8	Fold change: 0.290	Fold change: 0.047	Fold change: 0.755	Fold change: 4.556	Fold change: 0.314	Fold change: 1.324
	*p* = 0	*p* = 0	*p* = 0.026	*p* = 0.011	*p* = 0.07	*p* = 0.315
PIP + TET + ERY						
1	Fold change: 0.134	Fold change: 0.041	Fold change: 0.667	Fold change: 0.612	Fold change: 0.166	Fold change: 1.941
	*p* = NA	*p* = 0	*p* = 0.001	*p* = 0.002	*p* = 0.012	*p* = 0.048
2	Fold change: 0.659	Fold change: 0.086	Fold change: 1.035	Fold change: 0.221	Fold change: 0.073	Fold change: 1.502
	*p* = NA	*p* = 0	*p* = 0.831	*p* = 0	*p* = NA	*p* = 0.249
3	Fold change: 0.324	Fold change: 0.057	Fold change: 0.775	Fold change: 0.532	Fold change: 0.260	Fold change: 1.619
	*p* = 0	*p* = 0	*p* = 0.052	*p* = 0	*p* = 0.097	*p* = 0.618
4	Fold change: 0.601	Fold change: 0.060	Fold change: 0.737	Fold change: 0.589	Fold change: 0.559	Fold change: 1.350
	*p* = NA	*p* = 0	*p* = 0.024	*p* = 0	*p* = 0.698	*p* = 0.293
5	Fold change: 0.325	Fold change: 0.077	Fold change: 0.622	Fold change: 0.587	Fold change: 1.408	Fold change: 1.699
	*p* = NA	*p* = NA	*p* = 0	*p* = 0.004	*p* = 0.898	*p* = 0.186
6	Fold change: 0.600	Fold change: 0.081	Fold change: 0.830	Fold change: 0.616	Fold change: 10.437	Fold change: 1.119
	*p* = NA	*p* = 0	*p* = 0.18	*p* = 0.003	*p* = 0.727	*p* = 0.663
7	Fold change: 0.259	Fold change: 0.058	Fold change: 0.821	Fold change: 1.010	Fold change: 0.394	Fold change: 1.180
	*p* = 0	*p* = 0	*p* = 0.114	*p* = 0.963	*p* = 0.438	*p* = 0.516
8	Fold change: 0.271	Fold change: 0.099	Fold change: 1.660	Fold change: 1.895	Fold change: 0.547	Fold change: 1.611
	*p* = 0	*p* = 0	*p* = 0.032	*p* = 0.074	*p* = 0.714	*p* = 0.185
PIP + TET + NEO						
1	Fold change: 0.664	Fold change: 0.065	Fold change: 0.920	Fold change: 1.116	Fold change: 0.248	Fold change: 2.336
	*p* = 0	*p* = 0	*p* = 0.559	*p* = 0.635	*p* = 0.055	*p* = 0.037
2	Fold change: 0.595	Fold change: 0.053	Fold change: 0.821	Fold change: 1.323	Fold change: 0.241	Fold change: 1.130
	*p* = 0	*p* = 0	*p* = 0.129	*p* = 0.345	*p* = NA	*p* = 0.648
3	Fold change: 0.645	Fold change: 0.080	Fold change: 0.920	Fold change: 1.078	Fold change: 0.288	Fold change: 1.106
	*p* = 0	*p* = 0	*p* = 0.575	*p* = 0.724	*p* = 0.399	*p* = 0.739
4	Fold change: 0.213	Fold change: 0.089	Fold change: 0.904	Fold change: 1.013	Fold change: 0.273	Fold change: 1.205
	*p* = 0	*p* = 0	*p* = 0.479	*p* = 0.952	*p* = 0.399	*p* = 0.506
5	Fold change: 0.650	Fold change: 0.080	Fold change: 0.881	Fold change: 1.635	Fold change: 0.305	Fold change: 1.055
	*p* = 0.0003	*p* = 0	*p* = 0.325	*p* = 0.179	*p* = 0.419	*p* = NA
6	Fold change: 0.688	Fold change: 0.057	Fold change: 0.576	Fold change: 1.214	Fold change: 15.883	Fold change: 1.145
	*p* = NA	*p* = 0	*p* = 0	*p* = 0.437	*p* = 0.564	*p* = 0.652
7	Fold change: 0.626	Fold change: 0.082	Fold change: 0.781	Fold change: 1.053	Fold change: 0.150	Fold change: 1.577
	*p* = NA	*p* = 0	*p* = 0.050	*p* = 0.797	*p* = 0.011	*p* = 0.335
8	Fold change: 0.196	Fold change: 0.041	Fold change: 0.886	Fold change: 0.916	Fold change: 0.405	Fold change: 1.289
	*p* = 0	*p* = 0	*p* = 0.374	*p* = 0.661	*p* = 0.593	*p* = 0.414

**FIGURE 3:**
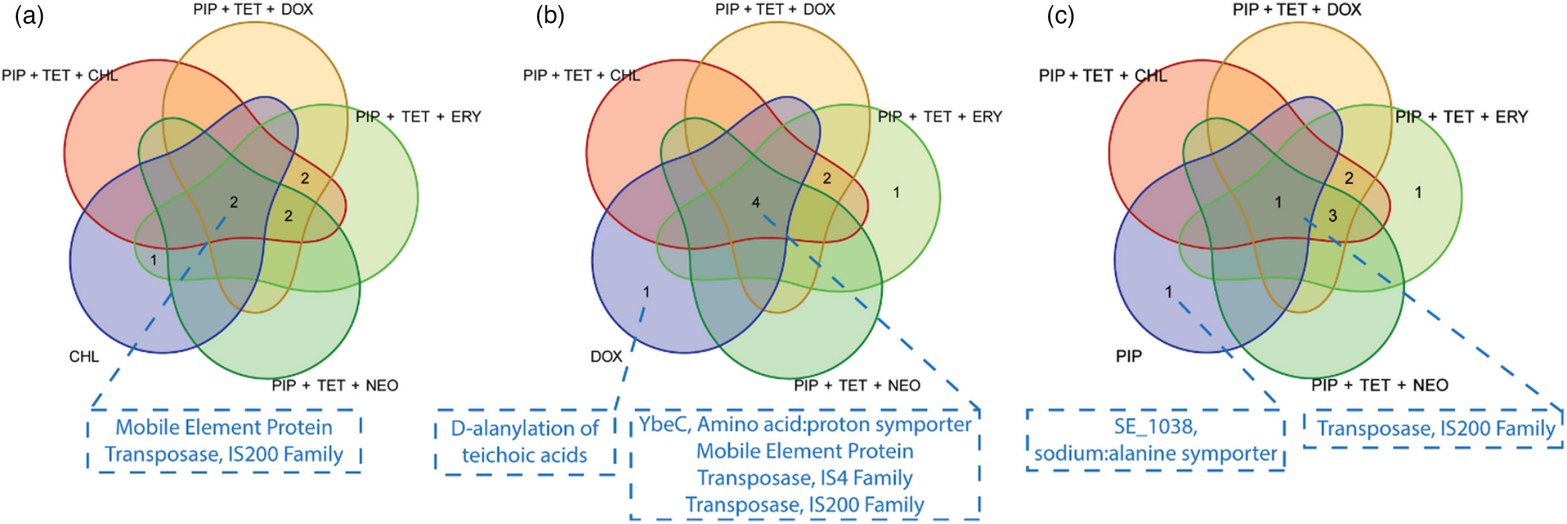
Overlap in highly conserved nonsynonymous genetic variant sets for synergistic drug combinations and collaterally sensitive (a) chloramphenicol‐resistant, (b) doxycycline‐resistant, and (c) piperacillin‐resistant strains. Missense mutations found in genes encoding mobile element protein and members of the IS200 transposase family were found in all drug combination‐resistant strains as well as the individual doxycycline‐resistant or chloramphenicol‐resistant strains. Furthermore, doxycycline resistance coincided with missense mutations in the amino acid‐proton symporter *YbeC* gene, and members of the IS4 transposase family, which were also found in all combination‐resistant strains. Overlap in nonsynonymous protein‐coding genetic variant sets for synergistic drug combinations and doxycycline resistance. Missense mutations found in genes encoding mobile element protein and the members of the IS4 and IS200 transposase families were conserved across all combination‐resistant and doxycycline‐resistant strains and coincide with the collateral sensitivity of *Staphylococcus epidermidis* to doxycycline. Mutations exclusive to the conserved doxycycline variant set include a 104G>A nucleotide point mutation, corresponding to a Gly35Asp substitution in the resulting D‐alanine‐poly (phosphoribitol) ligase, subunit one protein product.

We found that resistance to any of the combination regimens (PIP + TET + CHL, PIP + TET + DOX, PIP + TET + ERY, or PIP + TET + NEO) led to significant changes in the IC_50_ concentration of doxycycline or chloramphenicol. Nearly all strains resistant to any one of the combinations (PIP + TET + CHL, PIP + TET + DOX, PIP + TET + ERY, or PIP + TET + NEO) showed collateral sensitivity to doxycycline and chloramphenicol (Table 3, Figure 2). To explore potential mechanistic links between resistance to these combination regimens and the resulting observed collateral sensitivities, we used whole genome sequencing to identify highly conserved genetic variants that acquired nonsynonymous mutations independently in combination‐resistant, doxycycline‐resistant, and chloramphenicol‐resistant replicate sets (Figure S1A–F). Within both the combination and collaterally sensitive doxycycline and chloramphenicol strains, highly conserved missense mutations were found in genes that encode mobile element protein and members of the IS200 transposase family (Table 4, Figure 3a,b). Specifically, within a gene for a mobile element protein *SE0090*, missense 134T>C and 221A>G point mutations, corresponding to Leu45Ser and His74Arg amino acid substitutions were found among the combination and collaterally sensitive replicate sets, in addition to a dipeptide substitution at TyrGln66HisLys (Table 4). Within the *SE1292* gene, which encodes a member of the IS200 transposase family, a conserved 181G>A point mutation—corresponding to an Asp61Asn amino acid substitution—was also detected in the combination and collaterally sensitive replicate sets (Table 4). Overlap in mutations between the combination and doxycycline‐resistant sets was also observed in the amino acid/proton symporter *YbeC* gene and *SE0629*, which encodes a member of the IS4 transposase family (Table 4, Figure 3a). Within the *YbeC* gene, a 603G>C mutation and resulting Met201Ile substitution was observed, whereas *SE0629* experienced an 802G>A (Asp268Asn) substitution.

**TABLE 4:** Highly conserved nonsynonymous mutations across combinations and individual doxycycline‐resistant and chloramphenicol‐resistant *Staphylococcus epidermidis* strains.

Resistance	Associated gene/plasmid	Strains	Mutation type	Chromosomal position	Nucleotide substitution	Amino acid substitution
Piperacillin	*SE1292*	PIP: 1, 3–8	Missense	429	181G>A	Asp61Asn
		PIP: 1, 3–8	Missense	545	65T>C	Val22Ala
	*SE1038*	PIP: 3–5, 8	Frameshift/Del	272012	451delC	Leu151fs
		PIP: 6–7	Missense	272162	302G>A	Gly101Asp
Doxycycline	*SE0623*	DOX: 2–5, 8	Missense	326421	104G>A	Gly35Asp
	*YbeC*	DOX: 2–5, 8	Missense	107568	603G>C	Met201Ile
	*SE0090*	DOX: 2–5, 8	Missense	40114	221A>G	Hi74Arg
				40136	196TATC>CATA	TyrGln66HisLys
				40201	134T>C	Leu45Ser
	*SE0629*	DOX: 2–5, 8	Missense	754	802G>A	Asp268Asn
	*SE1292*	DOX: 2–5, 8	Missense	429	181G>A	Asp61Asn
		DOX: 2, 4–5, 8	Missense	545	65T>C	Val22Ala
Chloramphenicol	*SE0090*	CHL: 1–8	Missense	40114	221A>G	His74Arg
		CHL: 1–5, 7–8	Missense	40136	196TATC>CATA	TyrGln66HisLys
			Missense	40201	134T>C	Leu45Ser
	*SE1292*	CHL: 1–8	Missense	208	402G>T	Glu134Asp
		CHL: 2–8	Missense	545	65T>C	Val22Ala
	*SE0161*	CHL: 1–8	Missense	273	264AG>GA	GluGly88GluSer
PIP + TET + CHL	*YbeC*	PIP + TET + CHL: 1–8	Missense	107568	603G>C	Met201Ile
	*SE0691*	PIP + TET + CHL: 1, 3–5, 7–8	Stop Gained	116330	766G>T	Glu256*
		PIP + TET + CHL: 6	Stop Gained	116225	661G>T	Glu221*
		PIP + TET + CHL: 1–8	Missense	115658	94C>T	Pro32Ser
	*SE0090*	PIP + TET + CHL: 1–8	Missense	40114	221A>G	His74Arg
			Missense	40136	196TATC>CATA	TyrGln66HisLys
			Missense	40201	134T>C	Leu45Ser
	*SE0629*	PIP + TET + CHL: 1–8	Missense	754	802G>A	Asp268Asn
	*SE1292*	PIP + TET + CHL: 1–2, 4–8	Missense	429	181G>A	Asp61Asn
			Missense	545	65T>C	Val22Ala
		PIP + TET + CHL: 6	Missense	208	402G>T	Glu134Asp
	*SE0243*	PIP + TET + CHL: 1, 3–5, 7–8	Missense	45631	363C>G	Asp121Glu
PIP + TET + DOX	*YbeC*	PIP + TET + CHL: 1–8	Missense	107568	603G>C	Met201Ile
	*SE0691*	PIP + TET + CHL: 1–5, 6–8	Stop Gained	116330	766G>T	Glu256*
		PIP + TET + CHL: 5	Missense	115658	94C>	Pro32Ser
	*SE0090*	PIP + TET + CHL: 1–8	Missense	40114	221A>G	His74Arg
			Missense	40136	196TATC>CATA	TyrGln66HisLys
			Missense	40201	134T>C	Leu45Ser
	*SE0629*	PIP + TET + CHL: 1–8	Missense	754	802G>A	Asp268Asn
	*SE1292*	PIP + TET + CHL: 1–7	Missense	429	181G>A	Asp61Asn
			Missense	545	65T>C	Val22Ala
		PIP + TET + CHL: 8	Missense	754	802G>A	Asp268Asn
	*SE0243*	PIP + TET + CHL: 1–4, 6–8	Missense	45631	363C>G	Asp121Glu
PIP + TET + ERY	*YbeC*	PIP + TET + ERY: 1–8	Missense	107568	603G>C	Met201Ile
	*SE0691*	PIP + TET + ERY: 1–6	Stop Gained	116330	766G>T	Glu256*
		PIP + TET + ERY: 7	Missense	115943	379GC>AA	Ala127Asn
		PIP + TET + ERY: 7	Missense	115952	388G>T	Asp130Tyr
		PIP + TET + ERY: 8	Frameshift/Del	116060	503delT	Phe168fs
	*SE0090*	PIP + TET + ERY: 1–8	Missense	40114	221A>G	His74Arg
			Missense	40136	196TATC>CATA	TyrGln66HisLys
			Missense	40201	134T>C	Leu45Ser
	*SE0629*	PIP + TET + ERY: 1–8	Missense	754	802G>A	Asp268Asn
	*SE1292*	PIP + TET + ERY: 1–8	Missense	429	181G>A	Asp61Asn
			Missense	545	65T>C	Val22Ala
	*SE0243*	PIP + TET + ERY: 1–6	Missense	45631	363C>G	Asp121Glu
	*SE0161*	PIP + TET + ERY: 1–2, 4–6, 8	Missense	273	264AG>GA	GluGly88GluSer
PIP + TET + NEO	*SE0090*	PIP + TET + NEO: 1–8	Missense	40114	221A>G	His74Arg
			Missense	40136	196TATC>CATA	TyrGln66HisLys
			Missense	40201	134T>C	Leu45Ser
	*SE0629*	PIP + TET + NEO: 1–8	Missense	754	802G>A	Asp268Asn
	*SE1292*	PIP + TET + NEO: 1–8	Missense	429	181G>A	Asp61Asn
			Missense	545	65T>C	Val22Ala
	*YbeC*	PIP + TET + NEO: 1–4, 7–8	Missense	107568	603G>C	Met201Ile

*Note*: All mutations listed were found in ≥75% of the evaluated replicate sets and most mutations conferring missense variants or early termination in translation were consistently found in combination and doxycycline‐resistant and chloramphenicol‐resistant strains.

Piperacillin and tetracycline are both in all the combinations we studied (PIP + TET + CHL, PIP + TET + DOX, PIP + TET + ERY, and PIP + TET + NEO) yet susceptibility to piperacillin and tetracycline did not significantly change in strains that evolved resistance to these combinations (Figure 2, Table 3).

Collateral sensitivity to erythromycin was observed in three of the 32 combination‐resistant strains. One of the PIP + TET + DOX resistance strains showed cross‐resistance to neomycin and four strains of PIP + TET + ERY became collaterally sensitive to neomycin (Table 3, Figure 2, Table S1).

### 3.2 Resistance to single drugs leads to changes in susceptibility to combinations

**FIGURE 4:**
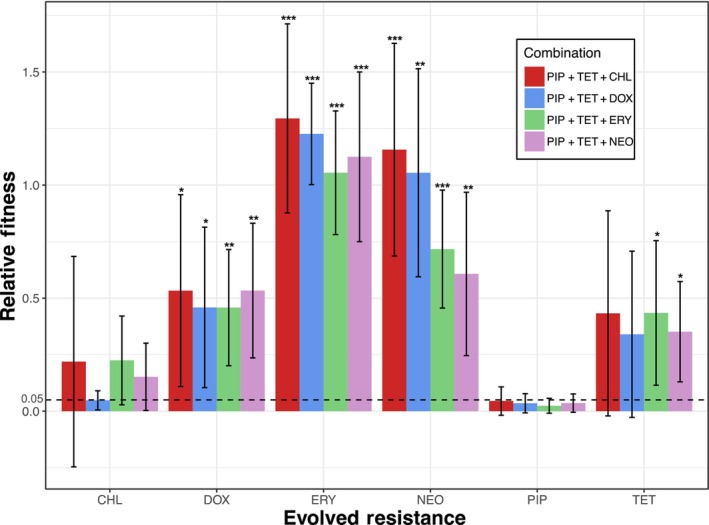
The evolution of resistance to some individual drug components results in a loss in susceptibility to the originally highly synergistic combination. The dashed line indicates a relative growth of 5%. Growth significantly higher than 5% gained some level of resistance to the combination. Otherwise, there was no significant impact on the strain's susceptibility to the highly synergistic combination. Error bars show 95% confidence intervals of the mean (*n* = 7). *p < 0.05, **p < 0.01, ***p < 0.001.

Evolving resistance to any single antibiotic typically resulted in an increase in resistance to the combinations to some degree (Table 5, Figure 4). Piperacillin‐resistant strains uniquely remained susceptible (i.e., not significantly greater than 5% relative growth) to all combinations—having the lowest estimated growth means compared to all other single‐drug resistant strains (Figure 4). We performed WGS for these strains to identify highly conserved nonsynonymous variant sets (Figure 3c, Figure S1G). Here, we detected conserved frameshift deletion and missense mutations in the *SE1038* gene, encoding the putative amino acid transporter (Table 4). Similar Asp61Asn and Val22Ala missense mutations from the IS200 transposase family member encoding *SE1292* gene were also detected here.

**TABLE 5:** Comparison of relative growth between ancestral strain and strains with evolved resistance to one of the single drug components.

Strain	Exposure to combination	Estimate	95% CI		*t*‐statistic	*p*‐value	*n*
			2.50%	97.50%			
CHL	PIP + TET + CHL	0.219	−0.246	0.685	0.860	0.418	7
DOX	PIP + TET + CHL	0.533	0.109	0.957	2.694	0.031	7
**ERY**	**PIP + TET + CHL**	**1.295**	**0.877**	**1.713**	**7.044**	**0.000**	**7**
**NEO**	**PIP + TET + CHL**	**1.157**	**0.686**	**1.627**	**5.565**	**0.001**	**7**
PIP	PIP + TET + CHL	0.045	−0.018	0.107	−0.195	0.851	7
TET	PIP + TET + CHL	0.433	−0.021	0.886	1.996	0.086	7
CHL	PIP + TET + DOX	0.048	0.006	0.090	−0.108	0.917	7
DOX	PIP + TET + DOX	0.459	0.104	0.814	2.726	0.030	7
**ERY**	**PIP + TET + DOX**	**1.226**	**1.002**	**1.451**	**12.844**	**0.000**	**6**
**NEO**	**PIP + TET + DOX**	**1.054**	**0.594**	**1.515**	**5.161**	**0.001**	**7**
PIP	PIP + TET + DOX	0.035	−0.007	0.078	−0.826	0.436	7
TET	PIP + TET + DOX	0.340	−0.027	0.708	1.931	0.102	6
CHL	PIP + TET + ERY	0.225	0.028	0.421	2.104	0.073	7
DOX	PIP + TET + ERY	0.458	0.201	0.715	3.759	0.007	7
**ERY**	**PIP + TET + ERY**	**1.054**	**0.781**	**1.328**	**8.694**	**0.000**	**7**
**NEO**	**PIP + TET + ERY**	**0.717**	**0.456**	**0.978**	**6.043**	**0.001**	**7**
PIP	PIP + TET + ERY	0.024	−0.009	0.057	−1.876	0.103	7
TET	PIP + TET + ERY	0.434	0.115	0.754	2.844	0.025	7
CHL	PIP + TET + NEO	0.152	0.003	0.301	1.623	0.149	7
DOX	PIP + TET + NEO	0.533	0.236	0.831	3.838	0.006	7
**ERY**	**PIP + TET + NEO**	**1.125**	**0.750**	**1.500**	**7.011**	**0.000**	**6**
NEO	PIP + TET + NEO	0.607	0.246	0.968	3.773	0.009	6
PIP	PIP + TET + NEO	0.036	−0.005	0.076	−0.824	0.437	7
TET	PIP + TET + NEO	0.351	0.130	0.573	3.213	0.015	7

*Note*: Significant differences after correcting for multiple tests (via Holm–Bonferroni, *α* = 0.0016) are shown in bold.

The chloramphenicol‐resistant strains were still susceptible to PIP + TET + DOX and were highly variable to PIP + TET + CHL, PIP + TET + ERY, and PIP + TET + NEO regimens. Tetracycline‐resistant strains showed mixed susceptibility and high variability when exposed to each combination. They remained susceptible to PIP + TET + CHL and PIP + TET + DOX but also showed decreased susceptibility to PIP + TET + ERY and PIP + TET + NEO. Doxycycline‐resistant strains showed less variation in growth than tetracycline‐resistant strains, yet relative growth did not change when exposed to any of the drug combinations (PIP + TET + CHL, PIP + TET + DOX, PIP + TET + ERY, or PIP + TET + NEO).

All other single antibiotic‐resistant strains (erythromycin‐resistant and neomycin‐resistant strains) led to the three‐drug combinations (PIP + TET + CHL, PIP + TET + DOX, PIP + TET + ERY, and PIP + TET + NEO) no longer being effective at suppressing growth. The erythromycin‐resistant and the neomycin‐resistant strains showed the overall strongest resistance to these combinations with some individual strains growing better in the presence of drug combinations than when antibiotics were absent. The strains resistant to a tetracycline‐class antibiotic—the tetracycline‐resistant strains and doxycycline‐resistant strains—showed high variability in growth when exposed to each combination. Although the mean values for the tetracycline‐resistant strains showed an increase in growth (over 5% relative growth), there was a large variation among each strain (Table 5, Figure 4).

### 3.3 Patterns of cross resistance and collateral sensitivity

**FIGURE 5:**
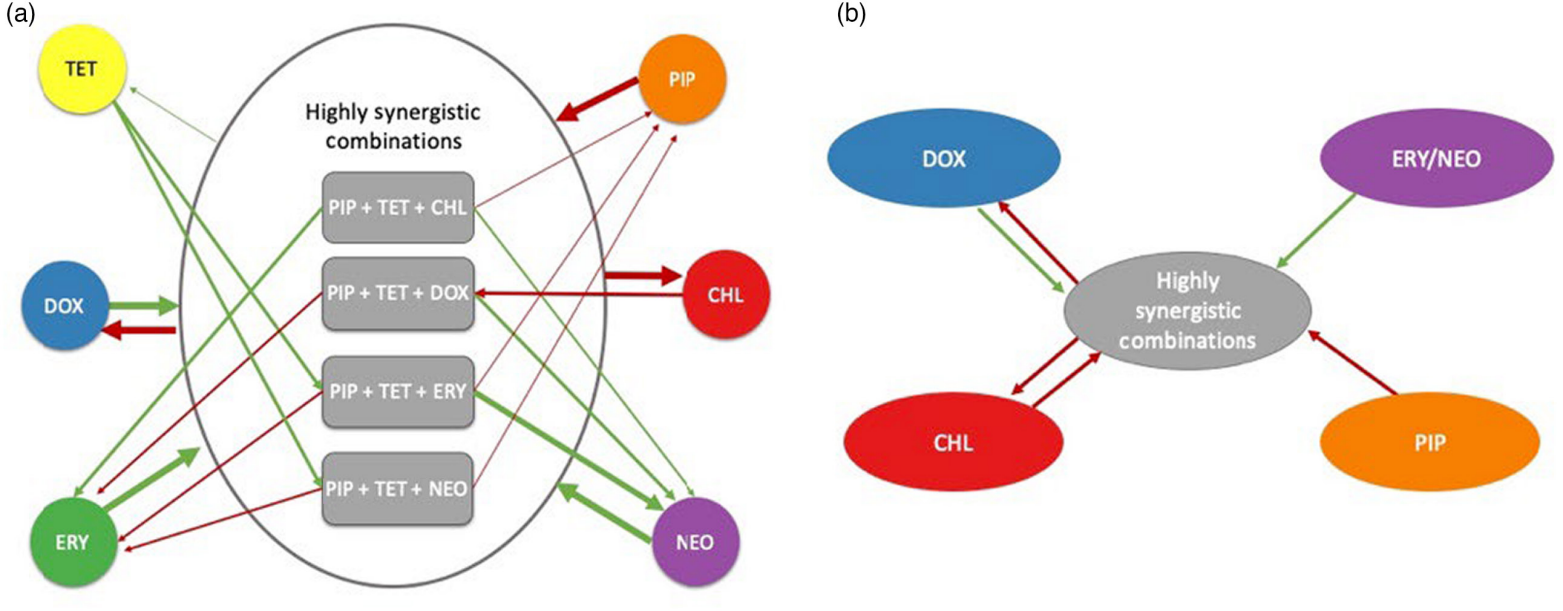
Patterns in the relationships between the four highly synergistic combinations and their individual components. Green arrows show a positive relationship for the bacteria: resistance to the combination/individual drug showed cross‐resistance or loss of sensitivity to the individual drug/combination. Red arrows show a negative relationship for the bacteria: resistance to the combination/individual drug showed collateral sensitivity or remained completely susceptible to the individual drug/combination. (a) Arrow weight shows how consistent the relationship is; the heavier the weight the more likely it is to observe the relationship. (b) Possible viable antibiotic sequence, resulting in less resistance evolving.

We examined patterns of the cross‐resistant and collateral sensitivity networks for all the evolved strains (Figure 5a). The cross‐resistant and collateral sensitivity networks can change among specific replicate populations and even show contrasting outcomes when evolving resistance to the same antibiotic or combination. Thus, we focus our attention on the trends we see among most or all of our independently evolved biological replicates (Figure 5b). Strain‐by‐strain fold changes in the MIC can be found in Figure 2a. For instance, despite the synergistic interaction between the antibiotic combinations, the collateral effects varied depending on the specific antibiotic. The relationships between piperacillin, doxycycline, chloramphenicol, and all the combinations tested in this study, are mostly consistent across a large majority of the independently evolved biological replicates (Figure 5b).

We also calculated the MICs of the single‐drug resistant strains to exposure to all other single drugs to consider the patterns of cross‐resistance and collateral sensitivities of single drug resistance among the single drugs (Figure S3). MIC values can be found in Figure S2. Resistance to any of the single drugs usually led to cross‐resistance to erythromycin. In contrast, resistance to any of the single drugs did not result in cross‐resistance—there was either no significant change in MIC or the strain became collaterally sensitive—to tetracycline. Collateral effects to piperacillin were mixed depending on the resistant strain (Figure S3).

### 3.4 Change in net and emergent interactions values

**FIGURE 6:**
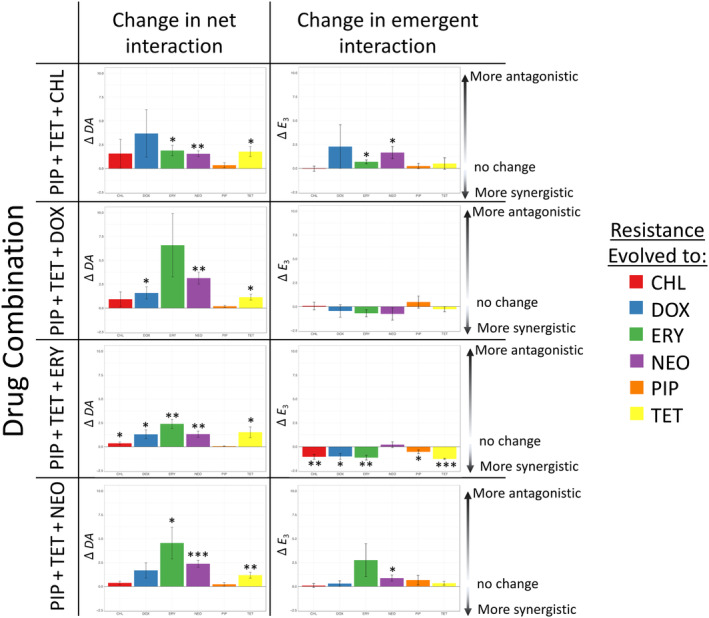
Net interactions are more likely than emergent interactions to be affected by the evolution of antibiotic resistance to a single antibiotic within the combination. Positive values indicate that the interaction is now more antagonistic with the evolved resistance than the ancestral strain. Negative values indicate that the interaction is now more synergistic with the evolved resistance than the ancestral strain (two‐tailed, one‐sample *t*‐test, *μ* = 0). Of the combinations tested, PIP + TET + ERY typically shows the most change in interaction values. *p < 0.05, **p < 0.01, ***p < 0.001.

Drug interactions were measured using both the ancestral strain and the evolved resistant strains to examine whether resistance to a single drug or resistance to the three‐drug combination changes the nature and effects of drug interactions. The interaction values of the combinations for the ancestral strain are listed in Table 2 and the degree of the change in the interaction values is shown in Figure 6 (interaction values for each strain for all two‐drug and three‐drug combinations can be found in Table S1).

We will first examine the net effects (*DA*), which are the overall effects that are due to all possible interactions within a combination. The net effects moved away from synergy and toward antagonism (the values became more positive) in cases that resulted in the three‐drug combinations no longer being effective at largely inhibiting growth. Figure 6 shows just how frequently the net effects significantly changed. Piperacillin‐resistant mutants were the only mutants to consistently have unchanged net effects (*DA* values). This means that the drug combinations remained highly synergistic (*p* < 0.05, two‐tailed, one‐sample *t*‐test, H_0_: *μ* = 0).

In contrast to the ever‐changing net effects (*DA*), the emergent effects (*E*_3_) were much more robust. The emergent effects are the effects of the interaction that is solely due to all three antibiotics being present in the combination. The emergent effects have fewer significant changes to the emergent interaction value (eight changes in the emergent effect vs. 14 changes in net effect out of a total possible 24 opportunities to change) (Figure 6). The changes in the emergent effect varied between becoming more antagonistic (positive) or synergistic (negative) with no clear trend. The combination of PIP + TET + ERY was the most susceptible to changes in interaction values, both net and emergent, effects when antibiotic resistance evolves.

When examining the strains resistant to the combinations themselves we saw the same trends as we saw with the single‐drug resistant strains. There was a shift toward antagonist values (more positive values). There were also more significant changes in the net effect than in the emergent effects (Figure S4, Table S1).

## 4 DISCUSSION

We show that evolving resistance to a combination of antibiotics results in varying types of collateral effects to the individual antibiotics within the combination (Figure 2). This suggests resistance to a combination is not only the result of a stepwise accumulation of mutations offering enhanced resistance to constituent antibiotics. Initially, predictive models for the evolution of resistance to a multidrug combination assumed that the mechanisms of resistance are independent of each other (Johnston et al., 2007; Komarova, 2006; Saputra et al., 2018). That is, a fully sensitive cell cannot become resistant to all drugs in the combination without undergoing multiple mutation events (Komarova, 2006). However, empirically it has been shown that these assumptions were not always supported in two‐drug combinations (Gifford et al., 2019). Work has been done to try to incorporate these findings into predictive evolutionary models (Berríos‐Caro et al., 2021). Furthermore, other factors such as tolerance—the ability for bacterial cells to survive but not actively grow in the presence of antibiotics—have also been shown to influence the predictions of these models (Liu et al., 2020). Even with the progress made and knowledge gained for two‐drug combinations, higher‐order combinations (3+ antibiotics) are not as well studied which is why they are the focus of our study. This study provides further empirical evidence to support the idea that the evolution of resistance to a combination, including higher‐order combinations, is not exclusively the result of a stepwise accumulation of resistance mutations to each antibiotic component within the combination.

Our results show that the evolution of resistance to a single component of a three‐drug combination does not always make the combination ineffective (Figure 4). These findings suggest that it could be crucial to consider combinations of drugs in addition to individual drugs when searching for a more viable antibiotic treatments. Others have also examined how the collateral effects of sequential treatment and the interaction between antibiotics relate for two‐drug combinations. Combinations that are made up of pairs that have the potential for collateral sensitivities tend to slow the rate of antibiotic resistance adaptations (Barbosa et al., 2018). Antagonistic combinations have been shown to slow rates of adaptation and result in resistance profiles that are different from those only resistant to one component of the combination (Dean et al., 2020). While synergistic combinations can have higher efficacy, they can also increase the selective advantage of resistance mutations over wild‐type strains (Torella et al., 2010).

In this study, we incorporated all components of the four 3‐drug combinations (piperacillin, tetracycline, chloramphenicol, doxycycline, erythromycin, neomycin) and the combinations themselves (PIP + TET + CHL, PIP + TET + DOX, PIP + TET + ERY, PIP + TET + NEO) to create a collateral effects network (Figure 4a). Using collateral effect networks—and even cellular hysteresis which has a much more rapid timescale (Roemhild et al., 2018)—to develop sequential/cyclical antibiotic treatments has been suggested as one approach to mitigate the problem of antibiotic resistance. In chronic infections such as cystic fibrosis, it may be effective to use phenotypes that, after initial treatment and the following evolved resistance, are still susceptible to other antibiotic options (Imamovic et al., 2018).

However, the success of using a collateral effect network to treat infections is often determined by the pair of antibiotics involved. Sequential use of antibiotics that make up a synergistic pair has been correlated with increased cross‐resistance between the two drugs (Fuentes‐Hernandez et al., 2015; Rodriguez de Evgrafov et al., 2015). However, using collateral sensitivities in principal may be leveraged in a clinical environment (Imamovic & Sommer, 2013). For example, sequential treatment of *Escherichia coli* with collaterally sensitive drug pairs can have higher efficacy at lower doses than using both antibiotics simultaneously (Fuentes‐Hernandez et al., 2015). In sequential treatments, collateral sensitives have even been shown to regenerate sensitivities to drug regimens that populations were previously resistant to (Barbosa et al., 2019; Dhawan et al., 2017). Antagonistic combinations have been shown to select against resistance adaptations when the bacteria has already adapted to one of the antibiotics within the combinations (Chait et al., 2007). Our findings suggest four potential three‐step treatments and four potential two‐step treatments where each step independently can result in collateral sensitivities.

In our examination of four possible three‐step treatments, we observed that if a bacterial population evolved resistance to piperacillin, it likely became susceptible to any one of the highly synergistic combinations, PIP + TET + CHL, PIP + TET + DOX, PIP + TET + ERY, or PIP + TET + NEO. The evolution of resistance to a combination leads to collateral sensitivity to doxycycline. The sequence of these treatments began with piperacillin, then any one of the combinations (PIP + TET + CHL, PIP + TET + DOX, PIP + TET + ERY, or PIP + TET + NEO), and ends with doxycycline. This order can promote collateral sensitivity as a population evolved resistance to each step of the sequence. But reversing the order, by evolving resistance to doxycycline first, resulted in the population becoming cross‐resistant to any of the combinations (PIP + TET + CHL, PIP + TET + DOX, PIP + TET + ERY, or PIP + TET + NEO).

Additionally, we investigated four treatments involving only two‐drug regimens: any one of the four 3‐drug combinations (PIP + TET + CHL, PIP + TET + DOX, PIP + TET + ERY, or PIP + TET + NEO) and chloramphenicol. Evolving resistance to any of these three‐drug combinations typically resulted in collateral sensitivities to chloramphenicol, and evolving resistance to chloramphenicol did not significantly influence susceptibility to the combinations. Thus, these treatments may continually select for collateral sensitivity to the other treatment in the pair.

We evaluated the collateral effect networks (Figure 5) assuming that: (1) there is little effect from epistatic interactions among the accumulated adaptive mutations as a population progresses through a sequence of antibiotics and (2) resistance adaptations give similar collateral effects. Epistasis has been identified as a mechanism enabling compensatory mutations to reduce the fitness cost of multidrug resistance (Das et al., 2020; Moura de Sousa et al., 2017) and resistance mutations for the same antibiotic can lead to varying collateral effects (Ardell & Kryazhimskiy, 2021; Maltas & Wood, 2019).

This study has possibly identified four two‐step treatments that are susceptible in both directions. That is, resistance to any one of four drug combinations tested in this study leads to higher susceptibility to chloramphenicol, and resistance to chloramphenicol still results in susceptibility to any of the synergistic drug combinations. Chloramphenicol resistance is typically mediated by monoacetylation and diacetylation by the chloramphenicol acetyltransferase enzyme. In staphylococci, two genes have been associated with this resistance—*cfr* and *fexA* (Kehrenberg & Schwarz, 2006). The gene *cfr* can mediate resistance to multiple antibiotics, such as clindamycin and florfenicol in addition to chloramphenicol (Kehrenberg et al., 2005). In contrast, the *fexA* gene represents a novel efflux protein that only accepts florfenicol and chloramphenicol (Kehrenberg & Schwarz, 2005).

All of the combinations examined in this study use tetracycline and piperacillin and a third antibiotic allowing for a characterization of the interaction between single‐drug resistance evolution and resistance evolution to these four synergistic combinations. Depending on (1) the type of drug regimen (a combination or an individual antibiotic) first driving populations to evolve resistance and (2) the order of subsequent exposures, the collateral effects show opposing trends where one direction promotes collateral sensitivity and the other promotes cross‐resistance (Figure 5b). For example, if a bacterial population evolves resistance to piperacillin, the collateral effect would not change the susceptibility to any one of the combinations—that is, there would be no collateral effect. The collateral effect of the evolution of resistance to a combination would be collateral sensitivity, in this case to doxycycline. This sequential order could promote continual sensitivity as a population evolves resistance to each step of the sequence. However, if the order is reversed—that is, evolving resistance to doxycycline first, then any one of the highly synergistic combinations, and ending with evolving resistance to piperacillin—the collateral effects of the sequence may lead to cross resistance across each step.

Let us first examine the mechanism of resistance evolution to doxycycline and piperacillin individually. Doxycycline is part of the tetracycline class, a family of antibiotics that inhibit protein synthesis. This is done by preventing the attachment of aminoacyl‐tRNA to the ribosomal acceptor (A) site (Chopra & Roberts, 2001). Evolving resistance to a tetracycline is a textbook example of the removal of the antibiotic through efflux pumps to keep intracellular concentrations low, rendering the antibiotic ineffective (Speer et al., 1992). This strategy has been associated with multidrug resistance across multiple classes due to the non‐specific nature of the efflux pump (Blanco et al., 2016; Webber & Piddock, 2003). However, the doxycycline‐resistant strains cultivated in this study have a mutation in one gene that is conserved that is unique to doxycycline resistance, *SE0623*. This gene codes for a component that is involved in the D‐alanylation of teichoic acids. The D‐alanylation of teichoic acids has been shown to provide some levels of antimicrobial peptide resistance in streptococcus (Kristian et al., 2005) and is a possible target pathway to inactivate in order to overcome multidrug resistance in MRSA (methicillin‐resistant *Staphylococcus aureus*) (Coupri et al., 2021). It is possible that evolving more general resistance mechanisms such as the D‐alanylation of teichoic acids may be enough to gain cross resistance to the highly synergistic combinations of this study: PIP + TET + CHL, PIP + TET + DOX, PIP + TET + ERY, PIP + TET + NEO (Figure 4).

Piperacillin is included in all combinations and is a part of the penicillin class, and is a β‐lactam. The main mechanism of resistance for this class is through the increased production of penicillin‐binding proteins (PBPs), proteins that are specific to β‐lactam resistance (Dever & Dermody, 1991). Piperacillin specifically evokes a paradox where it primarily selects for one variant of PBPs (PBP2b) even though its most reactive target is a different PBP (PBP2x). Both of these PBPs are essential and involved in peptidoglycan assembly resulting in resistance and leading to a change in morphology (Philippe et al., 2015). This change in morphology may account for some of the variability observed in the combination resistant strains, which are suspected of evolving piperacillin resistance (Figure 4), due to noise in optical density readings (Stevenson et al., 2016). Our results show that strains with evolved resistance to piperacillin both remained susceptible to the drug combinations and possessed a conserved mutation in genes *SE1292* and *SE1038*. This would suggest that the evolution of a more drug‐specific resistance adaptation, such as the PBPs selected for piperacillin resistance, is not enough to gain cross‐resistance to the synergistic combinations (Figure 5).

We show here that resistance to any of the antibiotic combinations in this study consistently results in collateral sensitivity to doxycycline and chloramphenicol. Whole genome sequencing revealed sets of highly conserved genetic variants that were found in combination‐resistant strains, and strains resistant to doxycycline and chloramphenicol, thus providing insight into potential evolutionary routes that *S. epidermidis* takes to acquire resistance. Here, highly conserved point mutations in genes encoding mobile genetic elements and transposases were found in combination‐resistant, doxycycline‐resistant, and chloramphenicol‐resistant strains (Table 4, Figure 3a,b), suggesting potential gene transfer mechanisms that are altered to overcome antibiotic stresses. Furthermore, within the doxycycline‐resistant and the combination‐resistant strains, which displayed the most consistent collateral sensitivity to doxycycline, conserved mutations in *YbeC* implicate a path toward resistance to the combinations involving serine/threonine metabolism. Importantly, these observations are made with the assumption that the mechanistic links between multidrug resistance and collateral sensitivity toward doxycycline and chloramphenicol exposure exist in protein‐coding regions of the *S. epidermidis* genome. Further investigation into non‐coding regions of the genome must be explicitly taken to rule out their roles in multidrug resistance.

Historically, net interactions (*DA*) have been the focus of evolutionary biologists (Chen et al., 2008; Cokol et al., 2017; Katzir et al., 2019; Zimmer et al., 2016) and ecologists (McCoy et al., 2012; Sitvarin & Rypstra, 2014; Sokol‐Hessner & Schmitz, 2002) because of the difficulties in determining and describing higher‐order emergent interactions accurately. Within the past 15 years, there has been substantial progress in determining and analyzing higher‐order interactions (Beppler et al., 2016, 2017; Cokol et al., 2017; Lozano‐Huntelman et al., 2021; Palmer et al., 2015; Tekin et al., 2016; Tekin, White, et al., 2018; Yeh et al., 2009; Zimmer et al., 2016). In both net and emergent interactions, the effects of an interaction are not primarily due to the chemical interactions between the compounds but rather due to how each individual component affects the physiology of the cell and how those effects interact (Bollenbach, 2015; Bollenbach et al., 2009; Mason et al., 2017). To add to the potential complexities of antibiotic interactions, interaction types may change when the concentration of the antibiotic changes, even if they are kept in the same ratios (Berenbaum et al., 1983), and a shift in specific dose combination can lead to different evolutionary outcomes (Gjini & Wood, 2021). Once the physiology of the cell is altered through evolved resistance adaptations, these interactions have the potential to change.

This study is the first to examine how both net and emergent interactions of drug combinations change in response to antibiotic resistance evolution. By testing synergistic combinations in both the ancestral susceptible strain and the single drug‐resistant strains, our results show that net interactions are more prone to change when a population evolves (i.e., adapted antibiotic resistance) (Figure 6). This makes the net interactions a more dynamic factor in response to the accumulation, loss, or change of physiological functions throughout the evolutionary history of a population. In contrast, emergent interactions appear to be more robust to these physiological changes and adaptations. Understanding how these emergent interactions can affect the evolutionary trajectory of populations will be key to creating long‐term plans to assess antibiotic resistance in natural and clinical populations. This is because although populations will continue to evolve and adapt to new and changing environments (Fitzgerald, 2019; Santos‐Lopez et al., 2019) the effects of emergent interactions may be more likely to persist due to their higher robustness, as shown in Figure 6. The ability to fine‐tune selection pressures to control evolutionary trajectories has been a goal for evolutionary biologists (Fischer et al., 2015; Iram et al., 2021) and we suggest that emergent interactions may be something to consider as a contributing factor.

In conclusion, we show that: (1) Evolving resistance to a combination of antibiotics being used simultaneously does not often lead to cross‐resistance to all of the components of that combination. (2) The evolution of resistance to one component of a combination does not always lead to cross‐resistance to the combination. (3) The evolution of resistance to a single antibiotic affects the net interaction more often than the emergent interaction of a combination. Our findings suggest that it is important to consider antibiotic combinations in addition to individual antibiotics when measuring and examining cross‐resistance and collateral sensitivity networks. Using those methods, we identified four possible two‐step treatments that confer susceptibility and collateral sensitivity to each other and four possible three‐step treatments where the sequence of the drug regimens can promote both types of collateral effects. This framework—examining both collateral sensitivity and cross‐resistance across whole networks of interactions at both the additive and emergent interaction levels—could allow researchers to uncover more viable sequential/cyclical treatment options that would extend the useful life span of antibiotics currently available. We encourage future studies to examine not just these possible treatments but to dive further into the genomic analysis to better identify possible antibiotic resistance genes that may be the target for these antibiotic combinations aside from those associated with each individual drug.

## CONFLICT OF INTEREST STATEMENT

The authors declare no conflicts of interest.

## Supporting information


Data S1

## Data Availability

The raw data will be available through Mendeley Data at https://data.mendeley.com/datasets/cfdk3y9skf/1.  Sequencing data is available via the National Center for Biotechnology Information under BioProject ID PRJNA1033048.
