# Supplementary material for: The evolution of resistance to synergistic multi‐drug combinations is more complex than evolving resistance to each individual drug component
Source: Evol Appl. 2023 Nov 15;16(12):1901–20. doi: 10.1111/eva.13608 (PMC10739078; doi:10.1111/eva.13608)
Supplement: Supplementary file 1 — Data S1 [file EVA-16-1901-s001.docx]

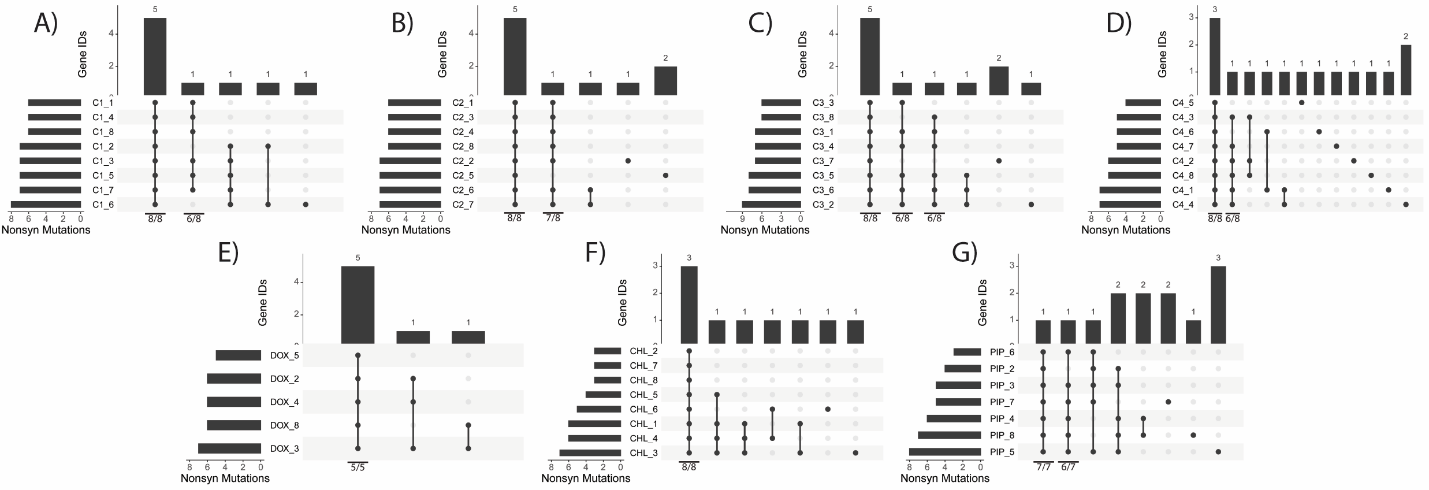


**Supplementary Figure 1.** **Upset plots depicting highly conserved nonsynonymous genetic variants amongst (A) PIP+TET+CHL, (B) PIP+TET+DOX, (C) PIP+TET+ERY, (D) PIP+TET+NEO, (E) DOX, (F) CHL, and (G) PIP replicate sets**. We define highly conserved using a threshold of ≥ 75%, indicating that a variant gene experienced a nonsynonymous mutation in at least 75% of the evaluated resistance strains.

**Supplementary Figure 2. MIC values of single drug-resistant strains**. All values greater than 2000 $\mu g$/mL were capped at 2000 $\mu g$/mL due to solubility issues with some antibiotics.

**A) B)**

**
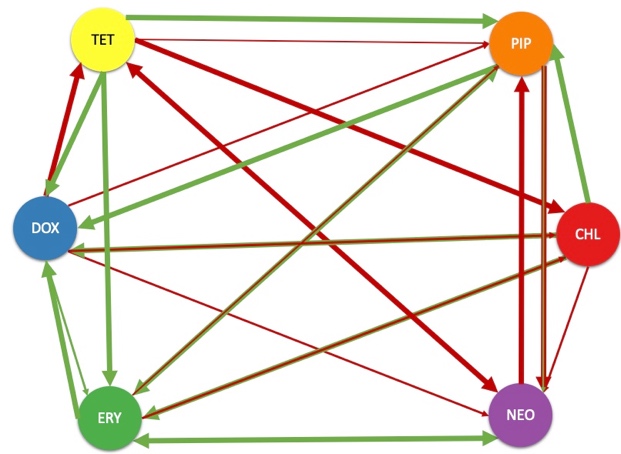

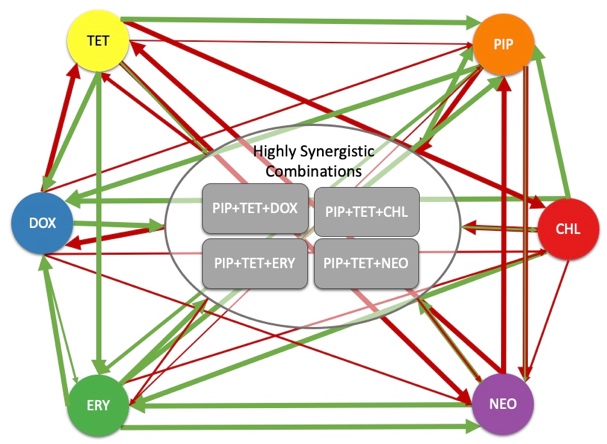
**

**Supplementary Figure 3. Collateral effect networks considering cross resistance and collateral sensitivity among single-drug resistant strains.** Green arrows show a positive relationship for the bacteria: resistance to the combination/individual drug showed cross-resistance or loss of sensitivity to the individual drug/combination. Red arrows show a negative relationship for the bacteria: resistance to the combination/individual drug showed collateral sensitivity or remained completely susceptible to the individual drug/combination. Arrow weight shows how consistent the relationship is; the heavier the weight the more likely it is to observe the relationship. A) Only considering single-drug resistant strains being exposed to other single drugs. B) Considering both exposures to single drugs and the highly synergistic three-drug combinations.

**Supplementary Figure 4.** **Net interactions are more likely than emergent interactions to be affected by the evolution of antibiotic resistance to a single ghly synergistic combination.** Positive values indicate that the interaction is now more antagonistic with the evolved resistance than the ancestral strain. Negative values indicate that the interaction is now more synergistic with the evolved resistance than the ancestral strain (two-tailed, one-sample t-test,$\mu$ = 0). Of the combinations tested, PIP+TET+ERY typically shows the most change in interaction values.

* p < 0.05, ** p < 0.01, *** p < 0.001

**Supplementary Table 1. Both raw and rescaled net (DA, rDA) and emergent (E3, rE3) interaction values for all combinations tested.** Please note that drug “A” is the first drug in the combination. Drug “B” is second and drug “C” is the third. So, for a combination with PIP+TET+CHL, PIP is drug “A”, TET is “B”, and CHL is “C”.

| **Combination** | **Evolved Resistance** | **Strain** | **DA (AB)** | **DA (AC)** | **DA (BC)** | **DA (ABC)** | **E3** | **rDA (AB)** | **rDA (AC)** | **rDA (BC)** | **rDA (ABC)** | **rE3** |
| --- | --- | --- | --- | --- | --- | --- | --- | --- | --- | --- | --- | --- |
| PIP+TET+CHL | CHL | CHL_1 | -0.402 | -0.034 | 0.010 | -0.381 | -0.001 | -0.936 | -0.048 | 0.224 | -0.966 | -0.001 |
| PIP+TET+CHL | CHL | CHL_2 | -0.437 | -0.285 | -0.266 | -0.419 | 0.352 | -0.866 | -0.372 | -0.563 | -0.979 | 0.879 |
| PIP+TET+CHL | CHL | CHL_3 | -0.136 | 0.317 | 0.186 | -0.069 | -0.185 | -0.920 | 1.640 | 1.230 | -0.864 | -1.529 |
| PIP+TET+CHL | CHL | CHL_4 | -0.509 | 0.145 | -0.015 | -0.463 | -0.078 | -0.914 | 2.140 | -0.025 | -0.903 | -0.089 |
| PIP+TET+CHL | CHL | CHL_5 | -0.151 | 0.122 | 0.023 | -0.128 | -0.108 | -0.540 | 0.554 | 0.128 | -0.775 | -0.502 |
| PIP+TET+CHL | CHL | CHL_6 | -0.168 | 0.023 | -0.123 | -0.536 | -0.288 | -0.262 | 0.448 | -0.206 | -0.951 | -0.496 |
| PIP+TET+CHL | CHL | CHL_7 | -0.486 | 0.111 | -0.076 | -0.559 | -0.157 | -0.721 | 2.263 | -0.126 | -0.983 | -0.189 |
| PIP+TET+CHL | CHL | CHL_8 | 0.626 | 0.031 | 0.479 | 1.217 | 0.574 | 2.761 | 0.152 | 3.143 | 11.133 | 0.952 |
| PIP+TET+CHL | Ancestral | Ancestral | -0.072 | 0.094 | -0.336 | -0.128 | -0.006 | -0.537 | 20.839 | -0.728 | -0.972 | -0.090 |
| PIP+TET+CHL | DOX | DOX_1 | 0.215 | -0.060 | -0.068 | 0.270 | 0.161 | 4.677 | -0.060 | -0.067 | 19.456 | 0.931 |
| PIP+TET+CHL | DOX | DOX_2 | -0.282 | 0.015 | -0.012 | -0.194 | 0.025 | -0.565 | 0.109 | -0.027 | -0.504 | 17.925 |
| PIP+TET+CHL | DOX | DOX_3 | 0.267 | -0.132 | -0.151 | -0.067 | -0.107 | 1.773 | -0.369 | -0.294 | -0.276 | -1.772 |
| PIP+TET+CHL | DOX | DOX_4 | 0.315 | 0.330 | 0.274 | 0.447 | -0.406 | 4.173 | 3.232 | 3.443 | 5.169 | -0.981 |
| PIP+TET+CHL | DOX | DOX_5 | -0.177 | 0.006 | 0.051 | -0.328 | -0.226 | -0.301 | 0.083 | 0.555 | -0.648 | -0.322 |
| PIP+TET+CHL | DOX | DOX_6 | -0.504 | -0.454 | -0.199 | -0.458 | 0.506 | -0.786 | -0.513 | -0.318 | -0.768 | 1.021 |
| PIP+TET+CHL | DOX | DOX_7 | -0.295 | 0.056 | 0.091 | -0.231 | -0.100 | -0.601 | 0.375 | 0.808 | -0.581 | -0.147 |
| PIP+TET+CHL | DOX | DOX_8 | -0.099 | -0.213 | -0.097 | -0.246 | 0.186 | -0.091 | -0.181 | -0.085 | -0.203 | 0.875 |
| PIP+TET+CHL | ERY | ERY_1 | 0.191 | 0.024 | 0.443 | 0.310 | -0.252 | 2.692 | 0.340 | 2.026 | 1.350 | -0.333 |
| PIP+TET+CHL | ERY | ERY_2 | -0.709 | -0.078 | -0.834 | -0.771 | 0.860 | -0.414 | -0.083 | -0.447 | -0.446 | 1.001 |
| PIP+TET+CHL | ERY | ERY_3 | -1.331 | -0.804 | -1.237 | -1.845 | 3.163 | -0.490 | -0.436 | -0.509 | -0.528 | 0.979 |
| PIP+TET+CHL | ERY | ERY_4 | 0.116 | -0.014 | -0.070 | 0.165 | 0.130 | 4.433 | -0.015 | -0.067 | 3.889 | 1.315 |
| PIP+TET+CHL | ERY | ERY_5 | -0.008 | -0.441 | -0.424 | -1.153 | 0.094 | -0.004 | -0.210 | -0.189 | -0.372 | 0.157 |
| PIP+TET+CHL | ERY | ERY_6 | 0.383 | 0.299 | 0.329 | 0.551 | 0.034 | 1.647 | 1.312 | 1.445 | 1.632 | 0.097 |
| PIP+TET+CHL | ERY | ERY_7 | 0.141 | 0.170 | 0.101 | 0.635 | 0.177 | 1.411 | 2.435 | 1.531 | 1.856 | 0.515 |
| PIP+TET+CHL | ERY | ERY_8 | 0.078 | -0.160 | -0.053 | -0.126 | 0.030 | 1.331 | -0.137 | -0.043 | -0.094 | 1.076 |
| PIP+TET+CHL | NEO | NEO_1 | 0.036 | -0.081 | 0.049 | 0.223 | 0.220 | 0.417 | -0.106 | 0.539 | 1.367 | 2.968 |
| PIP+TET+CHL | NEO | NEO_2 | 0.157 | 0.050 | 0.051 | 0.024 | -0.255 | 1.455 | 1.333 | 1.215 | 0.067 | -0.209 |
| PIP+TET+CHL | NEO | NEO_3 | -0.042 | -0.262 | -0.094 | -0.440 | 0.097 | -0.024 | -0.172 | -0.057 | -0.207 | 0.564 |
| PIP+TET+CHL | NEO | NEO_4 | 0.002 | -0.404 | -0.497 | -0.232 | 0.794 | 1.007 | -0.304 | -0.288 | -0.133 | 1.597 |
| PIP+TET+CHL | NEO | NEO_5 | 0.064 | -0.182 | -0.110 | 0.031 | 0.315 | 1.143 | -0.145 | -0.084 | 0.064 | 5.147 |
| PIP+TET+CHL | NEO | NEO_6 | 0.003 | 0.022 | -0.001 | 0.003 | 0.002 | 0.936 | 0.125 | -0.104 | 0.584 | 0.746 |
| PIP+TET+CHL | NEO | NEO_7 | 0.118 | -0.057 | -0.097 | 0.081 | 0.105 | 1.758 | -0.074 | -0.115 | 0.626 | 1.868 |
| PIP+TET+CHL | NEO | NEO_8 | 0.160 | 0.142 | 0.099 | 0.265 | -0.154 | 2.369 | 11.124 | 1.871 | 2.252 | -0.170 |
| PIP+TET+CHL | PIP | PIP_1 | 0.002 | 0.182 | 0.003 | 0.015 | -0.002 | 1.002 | 1.941 | 0.171 | 1.144 | -0.040 |
| PIP+TET+CHL | PIP | PIP_2 | -0.419 | -0.040 | -0.356 | -0.361 | 0.359 | -0.967 | -0.049 | -0.933 | -0.977 | 0.990 |
| PIP+TET+CHL | PIP | PIP_3 | -0.403 | 0.037 | -0.105 | -0.493 | -0.054 | -0.745 | 0.662 | -0.174 | -0.978 | -0.070 |
| PIP+TET+CHL | PIP | PIP_4 | -0.519 | 0.023 | -0.458 | -0.453 | 0.396 | -0.989 | 0.250 | -0.895 | -0.990 | 0.999 |
| PIP+TET+CHL | PIP | PIP_5 | 0.109 | 0.062 | 0.019 | -0.136 | -0.277 | 0.797 | 0.522 | 0.210 | -0.378 | -1.127 |
| PIP+TET+CHL | PIP | PIP_6 | -0.327 | 0.104 | -0.063 | -0.251 | -0.004 | -0.955 | 0.588 | -0.179 | -0.964 | -0.011 |
| PIP+TET+CHL | PIP | PIP_7 | -0.106 | 0.042 | -0.180 | -0.352 | -0.133 | -0.240 | 0.313 | -0.409 | -0.949 | -0.466 |
| PIP+TET+CHL | PIP | PIP_8 | -0.429 | 0.088 | -0.343 | -0.354 | 0.264 | -0.967 | 0.973 | -0.830 | -0.961 | 1.008 |
| PIP+TET+CHL | TET | TET_1 | -0.285 | -0.207 | -0.172 | -0.202 | 0.276 | -0.675 | -0.308 | -0.418 | -0.591 | 1.115 |
| PIP+TET+CHL | TET | TET_2 | 0.423 | -0.065 | -0.233 | 0.013 | -0.274 | 1.837 | -0.179 | -0.616 | 0.055 | -0.854 |
| PIP+TET+CHL | TET | TET_3 | 0.216 | 0.139 | 0.002 | 0.278 | 0.054 | 0.932 | 1.143 | 0.017 | 0.958 | 0.245 |
| PIP+TET+CHL | TET | TET_4 | 0.015 | -0.256 | -0.079 | 0.068 | 0.162 | 0.200 | -0.441 | -0.418 | 0.761 | 4.153 |
| PIP+TET+CHL | TET | TET_5 | 0.591 | 0.340 | 0.671 | 0.802 | -0.806 | 3.234 | 4.456 | 4.295 | 4.022 | -0.847 |
| PIP+TET+CHL | TET | TET_6 | 0.051 | 0.136 | 0.233 | 0.133 | -0.127 | 0.783 | 1.137 | 1.305 | 1.353 | -0.455 |
| PIP+TET+CHL | TET | TET_7 | -0.301 | -0.052 | -0.286 | -0.304 | 0.267 | -0.496 | -0.062 | -0.424 | -0.523 | 0.945 |
| PIP+TET+CHL | TET | TET_8 | 0.662 | 0.099 | 0.014 | 0.104 | -0.304 | 3.175 | 0.493 | 0.083 | 0.368 | -1.003 |
| PIP+TET+DOX | CHL | CHL_1 | -0.453 | -0.316 | 0.081 | -0.323 | 0.135 | -0.960 | -0.570 | 0.478 | -0.949 | 1.029 |
| PIP+TET+DOX | CHL | CHL_2 | -0.635 | -0.143 | -0.063 | -0.563 | 0.150 | -0.954 | -0.168 | -0.107 | -0.971 | 0.939 |
| PIP+TET+DOX | CHL | CHL_3 | -0.643 | -0.476 | -0.146 | -0.584 | 0.466 | -0.927 | -0.573 | -0.228 | -0.962 | 0.956 |
| PIP+TET+DOX | CHL | CHL_4 | -0.457 | 0.050 | -0.044 | -0.316 | 0.015 | -0.967 | 0.373 | -0.107 | -0.944 | 2.106 |
| PIP+TET+DOX | CHL | CHL_5 | -0.190 | 0.162 | 0.145 | -0.049 | -0.120 | -0.548 | 0.896 | 0.690 | -0.244 | -0.364 |
| PIP+TET+DOX | CHL | CHL_6 | -0.744 | -0.063 | -0.152 | -0.730 | 0.167 | -0.914 | -0.066 | -0.206 | -0.965 | 0.814 |
| PIP+TET+DOX | CHL | CHL_7 | -0.691 | 0.057 | 0.063 | -0.589 | -0.109 | -0.945 | 2.677 | 0.541 | -0.954 | -0.089 |
| PIP+TET+DOX | CHL | CHL_8 | -0.330 | 0.499 | -0.111 | 0.210 | 0.731 | -2.629 | 1.885 | -1.831 | 5.352 | 3.120 |
| PIP+TET+DOX | Ancestral | Ancestral | -0.421 | -0.698 | -0.167 | -0.318 | 0.466 | -0.999 | -0.964 | -0.507 | -1.001 | 0.998 |
| PIP+TET+DOX | DOX | DOX_1 | 0.281 | 0.252 | 0.136 | 0.436 | -0.130 | 4.350 | 2.535 | 1.617 | 2.811 | -0.757 |
| PIP+TET+DOX | DOX | DOX_2 | -0.217 | -0.118 | 0.093 | -0.145 | 0.020 | -0.466 | -0.214 | 0.576 | -0.421 | 4.242 |
| PIP+TET+DOX | DOX | DOX_3 | -0.044 | -0.003 | 0.113 | -0.218 | -0.280 | -0.088 | -0.005 | 0.667 | -0.602 | -0.571 |
| PIP+TET+DOX | DOX | DOX_4 | 0.038 | -0.006 | 0.077 | 0.325 | 0.223 | 1.112 | -0.006 | 1.834 | 3.771 | 2.065 |
| PIP+TET+DOX | DOX | DOX_5 | 0.016 | -0.112 | 0.128 | -0.237 | -0.290 | 0.202 | -0.174 | 0.779 | -0.584 | -0.504 |
| PIP+TET+DOX | DOX | DOX_6 | -0.366 | -0.007 | 0.158 | -0.324 | -0.188 | -0.527 | -0.009 | 1.356 | -0.595 | -0.190 |
| PIP+TET+DOX | DOX | DOX_7 | -0.414 | -0.238 | -0.038 | -0.324 | 0.200 | -0.786 | -0.358 | -0.072 | -0.752 | 1.080 |
| PIP+TET+DOX | DOX | DOX_8 | 0.277 | 0.200 | 0.270 | 0.340 | -0.183 | 1.352 | 1.113 | 1.549 | 1.032 | -1.014 |
| PIP+TET+DOX | ERY | ERY_1 | -0.498 | -0.494 | 0.018 | -0.530 | 0.535 | -0.287 | -0.300 | 1.086 | -0.285 | 1.056 |
| PIP+TET+DOX | ERY | ERY_2 | -0.133 | -0.223 | -0.792 | -0.490 | 0.733 | -0.099 | -0.194 | -0.447 | -0.296 | 1.515 |
| PIP+TET+DOX | ERY | ERY_3 | 0.485 | 0.321 | 0.059 | 0.408 | -0.519 | 3.213 | 6.935 | 1.324 | 1.841 | -1.213 |
| PIP+TET+DOX | ERY | ERY_4 | 0.060 | 0.350 | 0.066 | 0.184 | -0.277 | 2.136 | 13.998 | 2.210 | 3.523 | -0.483 |
| PIP+TET+DOX | ERY | ERY_5 | -15.572 | -28.159 | -28.649 | -152.851 | 228.273 | -0.698 | -0.829 | -0.831 | -0.946 | 0.925 |
| PIP+TET+DOX | ERY | ERY_6 | -0.035 | 0.058 | 0.406 | 0.106 | -0.326 | -0.035 | 10.532 | 3.978 | 22.475 | -0.264 |
| PIP+TET+DOX | ERY | ERY_7 | 0.155 | 0.200 | 0.202 | 0.724 | 0.225 | 9.696 | 16.054 | 1.637 | 12.644 | 0.584 |
| PIP+TET+DOX | ERY | ERY_8 | -2.635 | -0.801 | -4.349 | -5.557 | 6.552 | -0.701 | -0.419 | -0.727 | -0.847 | 0.892 |
| PIP+TET+DOX | NEO | NEO_1 | 0.057 | -0.064 | -0.010 | 0.148 | 0.165 | 2.123 | -0.087 | -0.012 | 1.890 | 3.714 |
| PIP+TET+DOX | NEO | NEO_2 | 0.271 | 0.234 | 0.282 | 0.362 | -0.326 | 5.796 | 3.807 | 2.035 | 3.729 | -0.653 |
| PIP+TET+DOX | NEO | NEO_3 | -0.809 | -0.105 | -0.464 | -1.504 | 0.470 | -0.329 | -0.053 | -0.186 | -0.433 | 0.565 |
| PIP+TET+DOX | NEO | NEO_4 | 0.348 | 0.071 | 0.118 | 0.359 | -0.134 | 6.267 | 2.011 | 2.059 | 4.055 | -0.334 |
| PIP+TET+DOX | NEO | NEO_5 | 0.095 | 0.079 | 0.055 | 0.249 | 0.016 | 3.965 | 3.598 | 1.996 | 4.510 | 0.088 |
| PIP+TET+DOX | NEO | NEO_6 | -0.005 | 1.111 | 0.007 | 0.008 | -0.013 | -0.434 | 2.121 | 0.925 | 0.978 | -2.751 |
| PIP+TET+DOX | NEO | NEO_7 | -0.124 | 0.078 | 0.031 | 0.122 | 0.134 | -0.147 | 1.086 | 0.420 | 0.868 | 1.329 |
| PIP+TET+DOX | NEO | NEO_8 | 0.151 | 0.044 | 0.063 | 0.165 | -0.104 | 2.439 | 1.633 | 1.594 | 1.497 | -0.104 |
| PIP+TET+DOX | PIP | PIP_1 | -0.007 | 0.038 | -0.011 | -0.005 | 0.010 | -0.125 | 2.696 | -0.217 | -0.101 | 2.435 |
| PIP+TET+DOX | PIP | PIP_2 | -0.326 | -0.448 | -0.267 | -0.239 | 0.414 | -0.966 | -0.663 | -0.960 | -0.947 | 1.012 |
| PIP+TET+DOX | PIP | PIP_3 | -0.320 | 0.048 | 0.051 | -0.217 | -0.057 | -0.969 | 0.246 | 0.353 | -0.961 | -0.124 |
| PIP+TET+DOX | PIP | PIP_4 | -0.408 | -0.503 | -0.313 | -0.311 | 0.517 | -0.974 | -0.755 | -0.842 | -0.967 | 1.005 |
| PIP+TET+DOX | PIP | PIP_5 | -0.526 | -0.030 | -0.014 | -0.298 | 0.170 | -0.960 | -0.041 | -0.028 | -0.655 | 5.431 |
| PIP+TET+DOX | PIP | PIP_6 | -0.374 | 0.000 | 0.023 | -0.272 | -0.014 | -0.971 | -0.001 | 0.177 | -0.959 | -0.025 |
| PIP+TET+DOX | PIP | PIP_7 | -0.538 | 0.005 | -0.134 | -0.437 | 0.131 | -0.982 | 0.124 | -0.281 | -0.967 | 1.055 |
| PIP+TET+DOX | PIP | PIP_8 | -0.478 | -0.438 | -0.227 | -0.380 | 0.450 | -0.965 | -0.604 | -0.514 | -0.953 | 1.011 |
| PIP+TET+DOX | TET | TET_1 | -0.320 | -0.285 | -0.101 | -0.175 | 0.280 | -0.717 | -0.522 | -0.270 | -0.580 | 1.173 |
| PIP+TET+DOX | TET | TET_2 | -0.330 | -0.253 | -0.029 | -0.194 | 0.170 | -0.819 | -0.482 | -0.088 | -0.739 | 1.140 |
| PIP+TET+DOX | TET | TET_3 | 0.177 | 0.170 | 0.213 | 0.256 | 0.069 | 0.844 | 0.862 | 1.237 | 0.940 | 0.518 |
| PIP+TET+DOX | TET | TET_5 | -0.051 | -0.119 | -0.242 | -0.106 | 0.331 | -0.043 | -0.111 | -0.184 | -0.082 | 1.661 |
| PIP+TET+DOX | TET | TET_6 | 0.000 | 0.014 | 0.130 | 0.110 | 0.015 | -0.004 | 0.255 | 0.858 | 0.680 | 0.157 |
| PIP+TET+DOX | TET | TET_7 | 0.094 | 0.146 | 0.189 | 0.361 | 0.146 | 0.586 | 0.776 | 0.886 | 1.252 | 0.856 |
| PIP+TET+DOX | TET | TET_8 | -0.196 | 0.079 | 0.166 | -0.116 | -0.164 | -0.521 | 0.512 | 0.784 | -0.528 | -0.393 |
| PIP+TET+ERY | CHL | CHL_1 | -0.549 | -0.525 | -0.041 | -0.557 | 0.421 | -0.972 | -0.674 | -0.055 | -0.969 | 1.004 |
| PIP+TET+ERY | CHL | CHL_2 | -0.208 | 0.039 | 0.013 | -0.442 | -0.285 | -0.337 | 0.754 | 0.334 | -0.761 | -0.374 |
| PIP+TET+ERY | CHL | CHL_3 | -0.526 | -0.084 | -0.028 | -0.511 | 0.071 | -0.992 | -0.095 | -0.051 | -1.000 | 0.949 |
| PIP+TET+ERY | CHL | CHL_4 | -0.569 | 0.020 | -0.002 | -0.553 | -0.032 | -0.943 | 0.692 | -0.004 | -0.982 | -0.030 |
| PIP+TET+ERY | CHL | CHL_5 | -0.042 | 0.062 | 0.056 | -0.447 | -0.504 | -0.063 | 1.088 | 1.175 | -0.703 | -0.738 |
| PIP+TET+ERY | CHL | CHL_6 | 0.054 | 0.183 | 0.094 | -0.039 | -0.313 | 1.811 | 4.710 | 1.299 | -0.060 | -0.555 |
| PIP+TET+ERY | CHL | CHL_7 | -0.025 | 0.059 | 0.035 | -0.102 | -0.153 | -0.039 | 0.796 | 0.605 | -0.172 | -0.251 |
| PIP+TET+ERY | CHL | CHL_8 | 0.003 | 0.048 | 0.077 | -0.362 | -0.476 | 0.136 | 1.648 | 1.269 | -0.523 | -0.654 |
| PIP+TET+ERY | Ancestral | Ancestral | -0.306 | -0.495 | -0.318 | -0.325 | 0.446 | -0.973 | -0.550 | -0.855 | -1.003 | 0.979 |
| PIP+TET+ERY | DOX | DOX_1 | 0.171 | 0.342 | 0.325 | 0.586 | 0.026 | 1.297 | 1.936 | 1.544 | 2.409 | 0.060 |
| PIP+TET+ERY | DOX | DOX_2 | -0.581 | 0.143 | -0.232 | -0.409 | 0.138 | -0.732 | 1.423 | -0.315 | -0.633 | 1.862 |
| PIP+TET+ERY | DOX | DOX_3 | 0.040 | 0.043 | 0.112 | -0.135 | -0.290 | 0.495 | 0.486 | 1.225 | -0.312 | -0.621 |
| PIP+TET+ERY | DOX | DOX_4 | 0.339 | 0.192 | 0.159 | 0.425 | -0.112 | 4.798 | 1.253 | 1.506 | 2.049 | -0.890 |
| PIP+TET+ERY | DOX | DOX_5 | -0.387 | 0.031 | 0.057 | -0.399 | -0.117 | -0.554 | 1.549 | 1.161 | -0.612 | -0.111 |
| PIP+TET+ERY | DOX | DOX_6 | -0.217 | -0.111 | -0.089 | -0.521 | -0.104 | -0.295 | -0.091 | -0.114 | -0.626 | -0.098 |
| PIP+TET+ERY | DOX | DOX_7 | -0.409 | 0.085 | 0.073 | -0.503 | -0.249 | -0.612 | 3.089 | 1.206 | -0.802 | -0.244 |
| PIP+TET+ERY | DOX | DOX_8 | 0.160 | 0.147 | 0.054 | 0.214 | -0.072 | 1.444 | 0.967 | 0.432 | 0.897 | -0.291 |
| PIP+TET+ERY | ERY | ERY_1 | 0.197 | -0.025 | -0.072 | 0.172 | 0.033 | 1.576 | -0.029 | -0.078 | 2.768 | 0.168 |
| PIP+TET+ERY | ERY | ERY_2 | 0.515 | 0.252 | 0.329 | 0.133 | -0.957 | 8.239 | 50.058 | 9.343 | 1.979 | -1.552 |
| PIP+TET+ERY | ERY | ERY_3 | 0.022 | -0.136 | 0.010 | -0.553 | -0.433 | 1.031 | -0.077 | 1.022 | -0.263 | -0.192 |
| PIP+TET+ERY | ERY | ERY_4 | 0.230 | 0.152 | 0.109 | 0.250 | -0.206 | 4.762 | 2.617 | 1.940 | 2.416 | -0.393 |
| PIP+TET+ERY | ERY | ERY_5 | -0.273 | -0.769 | -0.460 | -1.210 | 0.867 | -0.136 | -0.389 | -0.264 | -0.459 | 0.825 |
| PIP+TET+ERY | ERY | ERY_6 | 0.067 | 0.064 | 0.013 | 0.210 | 0.071 | 4.495 | 1.208 | 0.880 | 3.117 | 0.559 |
| PIP+TET+ERY | ERY | ERY_7 | 0.146 | 0.024 | 0.104 | 0.135 | -0.077 | 0.753 | 0.183 | 0.795 | 0.469 | -0.210 |
| PIP+TET+ERY | ERY | ERY_8 | 0.295 | 0.125 | 0.162 | 0.325 | -0.115 | 1.747 | 0.693 | 0.942 | 1.054 | -0.491 |
| PIP+TET+ERY | NEO | NEO_1 | 0.071 | 0.018 | -0.142 | 0.170 | 0.206 | 1.046 | 0.397 | -0.163 | 1.534 | 2.437 |
| PIP+TET+ERY | NEO | NEO_2 | 0.015 | 0.012 | 0.063 | 0.062 | -0.019 | 0.192 | 0.146 | 0.832 | 0.415 | -0.024 |
| PIP+TET+ERY | NEO | NEO_3 | -0.356 | -0.257 | -0.117 | -0.311 | 0.415 | -0.362 | -0.281 | -0.115 | -0.324 | 1.096 |
| PIP+TET+ERY | NEO | NEO_4 | 0.028 | -0.090 | -0.130 | -0.064 | 0.122 | 3.007 | -0.093 | -0.118 | -0.063 | 1.871 |
| PIP+TET+ERY | NEO | NEO_5 | 0.035 | 0.041 | -0.105 | 0.112 | 0.127 | 1.977 | 0.994 | -0.113 | 1.914 | 1.732 |
| PIP+TET+ERY | NEO | NEO_6 | -0.024 | 0.165 | -0.016 | -0.016 | 0.011 | -0.876 | 5.569 | -0.772 | -0.839 | 1.070 |
| PIP+TET+ERY | NEO | NEO_7 | -0.070 | -0.051 | -0.045 | -0.032 | 0.114 | -0.094 | -0.070 | -0.054 | -0.048 | 1.371 |
| PIP+TET+ERY | NEO | NEO_8 | -0.521 | 0.218 | 0.006 | -0.072 | -0.014 | -0.373 | 1.768 | 1.058 | -0.076 | -0.014 |
| PIP+TET+ERY | PIP | PIP_1 | -0.155 | -0.774 | -0.449 | -0.397 | 0.457 | -0.790 | -0.540 | -0.744 | -0.964 | 0.864 |
| PIP+TET+ERY | PIP | PIP_2 | -0.537 | 0.004 | 0.032 | -0.492 | -0.042 | -0.966 | 0.069 | 0.527 | -0.984 | -0.041 |
| PIP+TET+ERY | PIP | PIP_3 | -0.531 | -0.181 | 0.034 | -0.470 | 0.077 | -1.013 | -0.228 | 0.524 | -1.009 | 1.022 |
| PIP+TET+ERY | PIP | PIP_4 | -0.463 | 0.041 | -0.003 | -0.399 | -0.016 | -0.999 | 0.476 | -0.007 | -0.993 | -0.021 |
| PIP+TET+ERY | PIP | PIP_5 | -0.619 | -0.080 | -0.088 | -0.586 | 0.127 | -0.976 | -0.089 | -0.143 | -0.993 | 0.926 |
| PIP+TET+ERY | PIP | PIP_6 | -0.489 | 0.040 | 0.071 | -0.454 | -0.107 | -0.814 | 1.369 | 1.160 | -0.840 | -0.104 |
| PIP+TET+ERY | PIP | PIP_7 | -0.419 | 0.039 | 0.087 | -0.340 | -0.086 | -0.835 | 0.655 | 1.161 | -0.797 | -0.102 |
| PIP+TET+ERY | PIP | PIP_8 | -0.608 | -0.146 | -0.079 | -0.659 | 0.168 | -1.012 | -0.129 | -0.126 | -1.010 | 1.007 |
| PIP+TET+ERY | TET | TET_1 | -0.258 | 0.012 | 0.040 | -0.196 | 0.003 | -0.455 | 0.231 | 1.094 | -0.365 | 0.077 |
| PIP+TET+ERY | TET | TET_2 | -0.228 | 0.228 | 0.256 | -0.121 | -0.334 | -0.336 | 4.171 | 1.652 | -0.212 | -0.401 |
| PIP+TET+ERY | TET | TET_3 | 0.140 | 0.172 | 0.095 | 0.162 | -0.142 | 5.218 | 4.081 | 1.178 | 1.628 | -0.440 |
| PIP+TET+ERY | TET | TET_4 | -0.241 | 0.202 | 0.140 | -0.134 | -0.178 | -0.519 | 1.165 | 0.978 | -0.375 | -0.341 |
| PIP+TET+ERY | TET | TET_5 | 0.234 | 0.220 | 0.007 | 0.246 | -0.216 | 4.620 | 10.734 | 2.153 | 3.867 | -0.361 |
| PIP+TET+ERY | TET | TET_6 | 0.065 | 0.110 | 0.085 | 0.098 | -0.059 | 1.536 | 0.943 | 1.111 | 1.046 | -0.317 |
| PIP+TET+ERY | TET | TET_7 | -0.410 | 0.102 | 0.041 | -0.415 | -0.160 | -0.618 | 3.122 | 0.539 | -0.703 | -0.173 |
| PIP+TET+ERY | TET | TET_8 | -0.360 | 0.223 | 0.177 | -0.299 | -0.301 | -0.713 | 1.671 | 1.336 | -0.761 | -0.441 |
| PIP+TET+NEO | CHL | CHL_1 | -0.376 | 0.121 | 0.042 | -0.334 | -0.104 | -1.012 | 2.075 | 0.582 | -1.007 | -0.170 |
| PIP+TET+NEO | CHL | CHL_2 | -0.382 | 0.124 | 0.061 | -0.400 | -0.204 | -0.667 | 1.328 | 0.706 | -0.807 | -0.254 |
| PIP+TET+NEO | CHL | CHL_3 | -0.345 | 0.092 | -0.125 | -0.360 | 0.025 | -0.981 | 1.292 | -0.238 | -1.014 | 0.671 |
| PIP+TET+NEO | CHL | CHL_4 | -0.613 | 0.003 | -0.042 | -0.615 | 0.028 | -0.962 | 0.234 | -0.063 | -0.977 | 0.748 |
| PIP+TET+NEO | CHL | CHL_5 | -0.423 | -0.085 | -0.095 | -0.491 | 0.080 | -0.688 | -0.093 | -0.140 | -0.793 | 0.553 |
| PIP+TET+NEO | CHL | CHL_6 | -0.289 | -0.016 | 0.023 | -0.273 | -0.003 | -0.407 | -0.017 | 1.076 | -0.395 | -0.003 |
| PIP+TET+NEO | CHL | CHL_7 | -0.044 | 0.169 | 0.107 | -0.114 | -0.242 | -0.127 | 0.963 | 0.719 | -0.460 | -0.940 |
| PIP+TET+NEO | CHL | CHL_8 | 0.056 | 0.192 | 0.117 | 0.090 | -0.152 | 0.378 | 1.354 | 1.219 | 0.391 | -0.554 |
| PIP+TET+NEO | Ancestral | Ancestral | -0.196 | 0.065 | 0.006 | -0.153 | -0.023 | -1.003 | 0.476 | 0.083 | -1.001 | -0.080 |
| PIP+TET+NEO | DOX | DOX_1 | 0.003 | 0.144 | 0.011 | 0.127 | -0.014 | 0.029 | 1.739 | 0.141 | 0.818 | -0.029 |
| PIP+TET+NEO | DOX | DOX_2 | -0.217 | 0.240 | 0.113 | -0.110 | -0.179 | -0.434 | 1.868 | 0.847 | -0.282 | -0.353 |
| PIP+TET+NEO | DOX | DOX_3 | -0.288 | -0.255 | -0.290 | -0.300 | 0.379 | -0.563 | -0.299 | -0.536 | -0.618 | 0.935 |
| PIP+TET+NEO | DOX | DOX_4 | 0.080 | 0.045 | 0.012 | 0.342 | 0.213 | 2.258 | 1.388 | 1.179 | 6.122 | 1.812 |
| PIP+TET+NEO | DOX | DOX_5 | -0.047 | 0.019 | 0.009 | -0.262 | -0.235 | -0.068 | 1.298 | 1.033 | -0.362 | -0.306 |
| PIP+TET+NEO | DOX | DOX_6 | -0.349 | -0.123 | -0.082 | -0.653 | -0.050 | -0.402 | -0.090 | -0.091 | -0.632 | -0.034 |
| PIP+TET+NEO | DOX | DOX_7 | -0.576 | 0.458 | 0.099 | -0.153 | -0.307 | -0.752 | 7.163 | 1.195 | -0.349 | -0.670 |
| PIP+TET+NEO | DOX | DOX_8 | 0.149 | -0.054 | -0.007 | 0.142 | 0.052 | 0.935 | -0.080 | -0.010 | 0.706 | 0.393 |
| PIP+TET+NEO | ERY | ERY_1 | 0.152 | 0.104 | -0.106 | 0.198 | 0.026 | 1.261 | 1.484 | -0.076 | 0.329 | 0.087 |
| PIP+TET+NEO | ERY | ERY_2 | 0.123 | 0.484 | 0.411 | 0.422 | -0.476 | 1.200 | 25.717 | 3.946 | 3.537 | -0.883 |
| PIP+TET+NEO | ERY | ERY_3 | -1.311 | 0.023 | -0.225 | -0.968 | 0.921 | -0.367 | 1.018 | -0.112 | -0.240 | 2.237 |
| PIP+TET+NEO | ERY | ERY_4 | -0.066 | -0.026 | -0.003 | 0.089 | 0.185 | -0.058 | -0.029 | -0.003 | 0.705 | 5.407 |
| PIP+TET+NEO | ERY | ERY_5 | 0.178 | 0.048 | -0.122 | 0.188 | 0.053 | 7.777 | 1.343 | -0.119 | 8.161 | 0.224 |
| PIP+TET+NEO | ERY | ERY_6 | 0.101 | 0.028 | -0.016 | 0.372 | 0.260 | 11.904 | 0.848 | -0.015 | 11.036 | 2.047 |
| PIP+TET+NEO | ERY | ERY_7 | 0.003 | -0.015 | -0.206 | -0.027 | 0.126 | 0.042 | -0.022 | -0.236 | -0.045 | 11.809 |
| PIP+TET+NEO | ERY | ERY_8 | 0.129 | 0.058 | -0.002 | 0.164 | 0.000 | 1.958 | 0.609 | -0.003 | 1.066 | -0.001 |
| PIP+TET+NEO | NEO | NEO_2 | -0.079 | -0.096 | -0.214 | -0.024 | 0.378 | -0.071 | -0.094 | -0.188 | -0.021 | 2.009 |
| PIP+TET+NEO | NEO | NEO_3 | 0.144 | 0.121 | 0.140 | 0.236 | 0.025 | 0.665 | 0.590 | 0.596 | 0.722 | 0.174 |
| PIP+TET+NEO | NEO | NEO_4 | 0.171 | -0.053 | -0.074 | 0.194 | 0.138 | 3.257 | -0.066 | -0.081 | 2.719 | 1.183 |
| PIP+TET+NEO | NEO | NEO_5 | 0.377 | 0.150 | 0.187 | 0.483 | 0.166 | 1.689 | 0.710 | 0.802 | 1.528 | 0.663 |
| PIP+TET+NEO | NEO | NEO_6 | -0.002 | -0.021 | 0.003 | 0.005 | 0.004 | -0.344 | -0.040 | 1.003 | 1.421 | 1.607 |
| PIP+TET+NEO | NEO | NEO_7 | 0.210 | 0.205 | 0.218 | 0.279 | -0.003 | 0.889 | 0.889 | 0.902 | 0.822 | -0.416 |
| PIP+TET+NEO | NEO | NEO_8 | 0.237 | -0.122 | -0.101 | 0.151 | 0.061 | 1.604 | -0.138 | -0.110 | 2.405 | 0.322 |
| PIP+TET+NEO | PIP | PIP_1 | -0.048 | 0.197 | -0.121 | 0.101 | 0.101 | -0.768 | 1.882 | -0.820 | 0.513 | 3.726 |
| PIP+TET+NEO | PIP | PIP_2 | -0.360 | 0.192 | 0.230 | -0.276 | -0.278 | -0.935 | 1.023 | 1.373 | -0.961 | -0.442 |
| PIP+TET+NEO | PIP | PIP_3 | -0.595 | -0.086 | -0.091 | -0.599 | 0.151 | -0.988 | -0.088 | -0.142 | -0.972 | 1.071 |
| PIP+TET+NEO | PIP | PIP_4 | -0.473 | 0.361 | -0.008 | -0.432 | -0.185 | -0.984 | 5.067 | -0.015 | -0.976 | -0.274 |
| PIP+TET+NEO | PIP | PIP_5 | -0.460 | 0.032 | -0.019 | -0.514 | -0.078 | -0.840 | 0.734 | -0.033 | -0.985 | -0.085 |
| PIP+TET+NEO | PIP | PIP_6 | -0.354 | 0.118 | 0.047 | -0.526 | -0.299 | -0.566 | 7.498 | 1.124 | -0.856 | -0.321 |
| PIP+TET+NEO | PIP | PIP_7 | -0.563 | 0.042 | 0.012 | -0.531 | -0.033 | -0.989 | 0.881 | 0.402 | -0.983 | -0.032 |
| PIP+TET+NEO | PIP | PIP_8 | -0.537 | -0.121 | -0.086 | -0.606 | 0.154 | -0.987 | -0.101 | -0.148 | -0.986 | 1.005 |
| PIP+TET+NEO | TET | TET_1 | -0.590 | 0.091 | -0.376 | -0.500 | 0.274 | -0.732 | 1.113 | -0.445 | -0.685 | 1.144 |
| PIP+TET+NEO | TET | TET_2 | 0.089 | 0.119 | -0.208 | 0.106 | 0.033 | 0.624 | 3.834 | -0.335 | 0.648 | 0.204 |
| PIP+TET+NEO | TET | TET_3 | 0.019 | 0.157 | -0.022 | 0.095 | 0.021 | 0.170 | 0.897 | -0.058 | 0.479 | 0.233 |
| PIP+TET+NEO | TET | TET_4 | -0.376 | 0.067 | -0.025 | -0.326 | -0.002 | -0.693 | 0.936 | -0.047 | -0.662 | -0.002 |
| PIP+TET+NEO | TET | TET_5 | 0.099 | 0.017 | 0.173 | 0.301 | 0.049 | 1.256 | 0.163 | 1.766 | 1.740 | 0.209 |
| PIP+TET+NEO | TET | TET_6 | 0.071 | 0.136 | 0.050 | 0.077 | -0.061 | 1.074 | 1.039 | 1.066 | 0.738 | -0.467 |
| PIP+TET+NEO | TET | TET_7 | -0.424 | -0.257 | -0.297 | -0.421 | 0.456 | -0.667 | -0.264 | -0.470 | -0.672 | 0.993 |
| PIP+TET+NEO | TET | TET_8 | -0.086 | 0.189 | 0.003 | -0.049 | -0.068 | -0.276 | 1.734 | 0.035 | -0.189 | -0.276 |
| PIP+TET+CHL | PIP+TET+CHL | PIP+TET+CHL_1 | -0.287 | 0.091 | -0.036 | -0.211 | 0.025 | -0.557 | 1.793 | -0.087 | -0.483 | 3.629 |
| PIP+TET+DOX | PIP+TET+CHL | PIP+TET+CHL_1 | -0.220 | -0.598 | -0.227 | -0.227 | 0.525 | -0.484 | -0.724 | -0.382 | -0.480 | 1.282 |
| PIP+TET+ERY | PIP+TET+CHL | PIP+TET+CHL_1 | -0.006 | 0.248 | 0.398 | 0.062 | -0.361 | -0.031 | 5.388 | 1.475 | 0.912 | -0.760 |
| PIP+TET+NEO | PIP+TET+CHL | PIP+TET+CHL_1 | -0.736 | -0.003 | -0.005 | -0.726 | 0.023 | -0.587 | -0.002 | -0.005 | -0.575 | 2.559 |
| PIP+TET+CHL | PIP+TET+CHL | PIP+TET+CHL_2 | -0.778 | -0.160 | -0.293 | -0.734 | 0.535 | -0.736 | -0.122 | -0.398 | -0.726 | 1.018 |
| PIP+TET+DOX | PIP+TET+CHL | PIP+TET+CHL_2 | 0.326 | -0.174 | -0.179 | -0.037 | -0.191 | 7.156 | -0.398 | -0.177 | -0.097 | -1.997 |
| PIP+TET+ERY | PIP+TET+CHL | PIP+TET+CHL_2 | 0.327 | 0.196 | 0.679 | 0.465 | -0.571 | 1.446 | 2.119 | 1.834 | 10.790 | -0.881 |
| PIP+TET+NEO | PIP+TET+CHL | PIP+TET+CHL_2 | 0.009 | 0.142 | 0.027 | -0.032 | -0.185 | 1.064 | 4.601 | 1.135 | -0.038 | -0.247 |
| PIP+TET+CHL | PIP+TET+CHL | PIP+TET+CHL_3 | -0.570 | 0.107 | -0.259 | -0.449 | 0.228 | -0.695 | 2.079 | -0.389 | -0.640 | 1.204 |
| PIP+TET+DOX | PIP+TET+CHL | PIP+TET+CHL_3 | 0.119 | -0.169 | -0.094 | 0.052 | 0.054 | 0.691 | -0.403 | -0.156 | 0.338 | 0.580 |
| PIP+TET+ERY | PIP+TET+CHL | PIP+TET+CHL_3 | 0.126 | 0.196 | 0.334 | 0.203 | -0.230 | 2.594 | 1.690 | 1.430 | 1.715 | -0.725 |
| PIP+TET+NEO | PIP+TET+CHL | PIP+TET+CHL_3 | -0.110 | 0.165 | 0.134 | 0.021 | -0.158 | -0.126 | 7.115 | 1.706 | 0.201 | -0.181 |
| PIP+TET+CHL | PIP+TET+CHL | PIP+TET+CHL_4 | -0.390 | 0.011 | -0.111 | -0.333 | 0.112 | -0.676 | 0.138 | -0.194 | -0.641 | 1.194 |
| PIP+TET+DOX | PIP+TET+CHL | PIP+TET+CHL_4 | 0.039 | -0.200 | 0.057 | 0.021 | 0.049 | 0.199 | -0.497 | 1.116 | 0.117 | 0.773 |
| PIP+TET+ERY | PIP+TET+CHL | PIP+TET+CHL_4 | 0.218 | 0.162 | 0.364 | 0.308 | -0.167 | 6.009 | 0.933 | 1.415 | 4.827 | -0.922 |
| PIP+TET+NEO | PIP+TET+CHL | PIP+TET+CHL_4 | -0.213 | -0.029 | -0.040 | -0.178 | 0.113 | -0.269 | -0.026 | -0.052 | -0.212 | 1.734 |
| PIP+TET+CHL | PIP+TET+CHL | PIP+TET+CHL_5 | -0.577 | 0.026 | 0.044 | -0.450 | -0.030 | -0.740 | 1.170 | 0.355 | -0.687 | -0.026 |
| PIP+TET+DOX | PIP+TET+CHL | PIP+TET+CHL_5 | 0.109 | -0.480 | -0.019 | -0.268 | -0.022 | 0.776 | -0.655 | -0.020 | -0.478 | -0.029 |
| PIP+TET+ERY | PIP+TET+CHL | PIP+TET+CHL_5 | 0.337 | 0.043 | 0.511 | 0.464 | -0.283 | 17.243 | 0.490 | 1.605 | 9.856 | -0.920 |
| PIP+TET+NEO | PIP+TET+CHL | PIP+TET+CHL_5 | -0.423 | -0.167 | 0.117 | -0.386 | 0.061 | -0.367 | -0.125 | 3.492 | -0.319 | 21.133 |
| PIP+TET+CHL | PIP+TET+CHL | PIP+TET+CHL_6 | -0.556 | -0.040 | -0.294 | -0.485 | 0.335 | -0.729 | -0.046 | -0.403 | -0.696 | 1.073 |
| PIP+TET+DOX | PIP+TET+CHL | PIP+TET+CHL_6 | -0.060 | -0.329 | 0.043 | -0.064 | 0.193 | -0.151 | -0.580 | 1.127 | -0.166 | 5.793 |
| PIP+TET+ERY | PIP+TET+CHL | PIP+TET+CHL_6 | 0.297 | 0.224 | 0.498 | 0.523 | -0.064 | 5.468 | 1.048 | 1.566 | 5.710 | -0.351 |
| PIP+TET+NEO | PIP+TET+CHL | PIP+TET+CHL_6 | -0.128 | 0.132 | 0.132 | 0.092 | -0.033 | -0.146 | 3.336 | 2.067 | 1.219 | -0.034 |
| PIP+TET+CHL | PIP+TET+CHL | PIP+TET+CHL_7 | -0.479 | -0.022 | -0.190 | -0.422 | 0.201 | -0.731 | -0.027 | -0.285 | -0.706 | 1.080 |
| PIP+TET+DOX | PIP+TET+CHL | PIP+TET+CHL_7 | -0.095 | -0.238 | 0.056 | -0.057 | 0.128 | -0.371 | -0.605 | 0.646 | -0.262 | 2.370 |
| PIP+TET+ERY | PIP+TET+CHL | PIP+TET+CHL_7 | 0.291 | -0.048 | 0.440 | 0.518 | -0.224 | 1.379 | -0.045 | 1.579 | 32.214 | -0.564 |
| PIP+TET+NEO | PIP+TET+CHL | PIP+TET+CHL_7 | 0.122 | 0.123 | 0.097 | 0.328 | 0.068 | 0.890 | 0.938 | 0.887 | 1.466 | 0.371 |
| PIP+TET+CHL | PIP+TET+CHL | PIP+TET+CHL_8 | -0.469 | 0.002 | -0.187 | -0.382 | 0.201 | -0.709 | 0.038 | -0.309 | -0.665 | 1.141 |
| PIP+TET+DOX | PIP+TET+CHL | PIP+TET+CHL_8 | -0.199 | -0.393 | 0.064 | -0.144 | 0.206 | -0.510 | -0.660 | 0.842 | -0.428 | 1.612 |
| PIP+TET+ERY | PIP+TET+CHL | PIP+TET+CHL_8 | 0.050 | 0.026 | 0.365 | 0.242 | -0.172 | 45.125 | 5.636 | 1.453 | 11.516 | -0.340 |
| PIP+TET+NEO | PIP+TET+CHL | PIP+TET+CHL_8 | -0.144 | 0.168 | 0.177 | 0.228 | 0.098 | -0.305 | 1.000 | 1.330 | 0.936 | 0.410 |
| PIP+TET+CHL | PIP+TET+DOX | PIP+TET+DOX_1 | -0.496 | -0.039 | -0.124 | -0.466 | 0.161 | -0.711 | -0.040 | -0.189 | -0.698 | 1.057 |
| PIP+TET+DOX | PIP+TET+DOX | PIP+TET+DOX_1 | -0.103 | -0.231 | -0.012 | -0.063 | 0.175 | -0.326 | -0.541 | -0.022 | -0.235 | 1.877 |
| PIP+TET+ERY | PIP+TET+DOX | PIP+TET+DOX_1 | 0.169 | -0.228 | 0.488 | 0.163 | -0.562 | 1.234 | -0.167 | 1.664 | 1.884 | -0.734 |
| PIP+TET+NEO | PIP+TET+DOX | PIP+TET+DOX_1 | -0.119 | -0.189 | -0.062 | -0.241 | 0.084 | -0.169 | -0.161 | -0.083 | -0.306 | 0.437 |
| PIP+TET+CHL | PIP+TET+DOX | PIP+TET+DOX_2 | -0.728 | -0.205 | -0.248 | -0.702 | 0.473 | -0.745 | -0.173 | -0.318 | -0.738 | 1.014 |
| PIP+TET+DOX | PIP+TET+DOX | PIP+TET+DOX_2 | -0.159 | -0.305 | -0.192 | -0.167 | 0.330 | -0.393 | -0.552 | -0.247 | -0.401 | 1.237 |
| PIP+TET+ERY | PIP+TET+DOX | PIP+TET+DOX_2 | -0.025 | -0.012 | 0.485 | 0.097 | -0.397 | -0.109 | -0.012 | 1.600 | 10.773 | -0.531 |
| PIP+TET+NEO | PIP+TET+DOX | PIP+TET+DOX_2 | -0.362 | -0.311 | -0.123 | -0.500 | 0.326 | -0.401 | -0.217 | -0.131 | -0.453 | 0.850 |
| PIP+TET+CHL | PIP+TET+DOX | PIP+TET+DOX_3 | -0.640 | -0.013 | -0.230 | -0.546 | 0.266 | -0.733 | -0.015 | -0.295 | -0.701 | 1.103 |
| PIP+TET+DOX | PIP+TET+DOX | PIP+TET+DOX_3 | -0.245 | -0.432 | -0.105 | -0.251 | 0.387 | -0.507 | -0.644 | -0.139 | -0.506 | 1.213 |
| PIP+TET+ERY | PIP+TET+DOX | PIP+TET+DOX_3 | 0.025 | 0.033 | 0.542 | 0.185 | -0.342 | 1.537 | 0.465 | 1.660 | 4.690 | -0.540 |
| PIP+TET+NEO | PIP+TET+DOX | PIP+TET+DOX_3 | -0.197 | -0.126 | -0.085 | -0.315 | 0.101 | -0.207 | -0.111 | -0.088 | -0.307 | 0.494 |
| PIP+TET+CHL | PIP+TET+DOX | PIP+TET+DOX_4 | -0.358 | 0.007 | -0.130 | -0.296 | 0.129 | -0.688 | 0.069 | -0.251 | -0.648 | 1.166 |
| PIP+TET+DOX | PIP+TET+DOX | PIP+TET+DOX_4 | -0.059 | -0.173 | -0.027 | -0.026 | 0.138 | -0.249 | -0.495 | -0.052 | -0.126 | 2.226 |
| PIP+TET+ERY | PIP+TET+DOX | PIP+TET+DOX_4 | -0.637 | -2.010 | -0.017 | -1.500 | 0.739 | -0.627 | -0.548 | -0.018 | -0.800 | 0.696 |
| PIP+TET+NEO | PIP+TET+DOX | PIP+TET+DOX_4 | -0.302 | -0.053 | 0.000 | -0.308 | 0.019 | -0.593 | -0.054 | 0.023 | -0.610 | 0.684 |
| PIP+TET+CHL | PIP+TET+DOX | PIP+TET+DOX_5 | -0.527 | -0.086 | -0.273 | -0.440 | 0.382 | -0.710 | -0.089 | -0.454 | -0.670 | 1.073 |
| PIP+TET+DOX | PIP+TET+DOX | PIP+TET+DOX_5 | 0.074 | -0.155 | 0.047 | 0.004 | 0.003 | 0.441 | -0.388 | 1.110 | 0.026 | 0.036 |
| PIP+TET+ERY | PIP+TET+DOX | PIP+TET+DOX_5 | -0.432 | -1.528 | -0.080 | -0.993 | 0.542 | -0.493 | -0.474 | -0.113 | -0.698 | 0.650 |
| PIP+TET+NEO | PIP+TET+DOX | PIP+TET+DOX_5 | -0.088 | -0.070 | 0.022 | -0.084 | 0.004 | -0.211 | -0.068 | 1.035 | -0.211 | 1.059 |
| PIP+TET+CHL | PIP+TET+DOX | PIP+TET+DOX_6 | -0.432 | 0.134 | -0.208 | -0.323 | 0.120 | -0.690 | 1.292 | -0.351 | -0.625 | 1.392 |
| PIP+TET+DOX | PIP+TET+DOX | PIP+TET+DOX_6 | -0.056 | -0.196 | -0.127 | -0.095 | 0.166 | -0.186 | -0.478 | -0.156 | -0.298 | 1.527 |
| PIP+TET+ERY | PIP+TET+DOX | PIP+TET+DOX_6 | -0.163 | -0.936 | 0.087 | -0.538 | -0.073 | -0.288 | -0.382 | 1.206 | -0.602 | -0.045 |
| PIP+TET+NEO | PIP+TET+DOX | PIP+TET+DOX_6 | -0.236 | 0.014 | -0.044 | -0.221 | 0.038 | -0.414 | 0.437 | -0.074 | -0.402 | 1.205 |
| PIP+TET+CHL | PIP+TET+DOX | PIP+TET+DOX_7 | -0.029 | 0.021 | -0.041 | -0.303 | -0.259 | -0.058 | 0.251 | -0.069 | -0.670 | -0.598 |
| PIP+TET+DOX | PIP+TET+DOX | PIP+TET+DOX_7 | -0.041 | 0.024 | 0.053 | -0.208 | -0.231 | -0.081 | 0.211 | 0.625 | -0.480 | -0.465 |
| PIP+TET+ERY | PIP+TET+DOX | PIP+TET+DOX_7 | -0.256 | -0.810 | 0.305 | -0.551 | -0.293 | -0.441 | -0.378 | 1.745 | -0.644 | -0.146 |
| PIP+TET+NEO | PIP+TET+DOX | PIP+TET+DOX_7 | -0.287 | 0.011 | 0.018 | -0.227 | 0.021 | -0.615 | 0.206 | 0.580 | -0.517 | 0.939 |
| PIP+TET+CHL | PIP+TET+DOX | PIP+TET+DOX_8 | -0.008 | -0.006 | -0.101 | -0.158 | -0.068 | -0.014 | -0.009 | -0.168 | -0.337 | -0.171 |
| PIP+TET+DOX | PIP+TET+DOX | PIP+TET+DOX_8 | 0.069 | 0.034 | -0.012 | -0.127 | -0.199 | 0.507 | 0.344 | -0.022 | -0.293 | -0.581 |
| PIP+TET+ERY | PIP+TET+DOX | PIP+TET+DOX_8 | -0.102 | -0.630 | 0.170 | -0.267 | -0.177 | -0.227 | -0.348 | 1.286 | -0.463 | -0.154 |
| PIP+TET+NEO | PIP+TET+DOX | PIP+TET+DOX_8 | -0.179 | -0.018 | -0.058 | -0.184 | 0.054 | -0.298 | -0.020 | -0.094 | -0.318 | 0.820 |
| PIP+TET+CHL | PIP+TET+ERY | PIP+TET+ERY_1 | 0.032 | -0.003 | 0.083 | -0.096 | -0.197 | 0.653 | -0.004 | 1.145 | -0.249 | -0.455 |
| PIP+TET+DOX | PIP+TET+ERY | PIP+TET+ERY_1 | -0.023 | 0.006 | 0.087 | -0.022 | -0.080 | -0.054 | 0.050 | 1.151 | -0.059 | -0.172 |
| PIP+TET+ERY | PIP+TET+ERY | PIP+TET+ERY_1 | -0.384 | -2.637 | 0.250 | -1.744 | -0.179 | -0.369 | -0.586 | 9.112 | -0.794 | -0.037 |
| PIP+TET+NEO | PIP+TET+ERY | PIP+TET+ERY_1 | -0.158 | 0.044 | 0.049 | -0.142 | -0.056 | -0.355 | 1.122 | 1.095 | -0.335 | -0.095 |
| PIP+TET+CHL | PIP+TET+ERY | PIP+TET+ERY_2 | -0.161 | -0.077 | 0.002 | -0.209 | -0.021 | -0.226 | -0.085 | 0.024 | -0.341 | -0.026 |
| PIP+TET+DOX | PIP+TET+ERY | PIP+TET+ERY_2 | -0.268 | -0.035 | 0.017 | -0.202 | 0.037 | -0.414 | -0.040 | 0.191 | -0.360 | 5.833 |
| PIP+TET+ERY | PIP+TET+ERY | PIP+TET+ERY_2 | -0.712 | -1.789 | -0.077 | -1.860 | 0.742 | -0.558 | -0.512 | -0.065 | -0.812 | 0.561 |
| PIP+TET+NEO | PIP+TET+ERY | PIP+TET+ERY_2 | 0.012 | -0.044 | -0.044 | -0.125 | -0.073 | 0.275 | -0.043 | -0.071 | -0.219 | -0.134 |
| PIP+TET+CHL | PIP+TET+ERY | PIP+TET+ERY_3 | 0.008 | -0.045 | 0.068 | -0.180 | -0.216 | 0.204 | -0.055 | 0.641 | -0.276 | -0.289 |
| PIP+TET+DOX | PIP+TET+ERY | PIP+TET+ERY_3 | -0.136 | -0.036 | 0.056 | -0.124 | -0.028 | -0.191 | -0.047 | 0.495 | -0.203 | -0.035 |
| PIP+TET+ERY | PIP+TET+ERY | PIP+TET+ERY_3 | -0.321 | -2.082 | 0.393 | -1.617 | -0.599 | -0.296 | -0.541 | 5.246 | -0.757 | -0.128 |
| PIP+TET+NEO | PIP+TET+ERY | PIP+TET+ERY_3 | -0.102 | -0.036 | 0.028 | -0.101 | -0.002 | -0.171 | -0.039 | 1.079 | -0.170 | -0.002 |
| PIP+TET+CHL | PIP+TET+ERY | PIP+TET+ERY_4 | -0.055 | 0.035 | 0.009 | -0.182 | -0.160 | -0.129 | 0.307 | 0.120 | -0.496 | -0.397 |
| PIP+TET+DOX | PIP+TET+ERY | PIP+TET+ERY_4 | 0.051 | 0.021 | 0.014 | 0.023 | -0.045 | 0.458 | 0.228 | 0.226 | 0.141 | -0.128 |
| PIP+TET+ERY | PIP+TET+ERY | PIP+TET+ERY_4 | -0.207 | -1.300 | 0.087 | -0.609 | 0.071 | -0.365 | -0.481 | 1.222 | -0.630 | 0.329 |
| PIP+TET+NEO | PIP+TET+ERY | PIP+TET+ERY_4 | -0.167 | -0.008 | 0.052 | -0.139 | -0.028 | -0.440 | -0.011 | 1.088 | -0.402 | -0.051 |
| PIP+TET+CHL | PIP+TET+ERY | PIP+TET+ERY_5 | -0.010 | -0.014 | -0.012 | -0.110 | -0.081 | -0.016 | -0.018 | -0.021 | -0.209 | -0.152 |
| PIP+TET+DOX | PIP+TET+ERY | PIP+TET+ERY_5 | -0.025 | 0.042 | 0.034 | -0.036 | -0.070 | -0.048 | 0.511 | 0.373 | -0.084 | -0.155 |
| PIP+TET+ERY | PIP+TET+ERY | PIP+TET+ERY_5 | -0.063 | -0.525 | 0.132 | -0.319 | -0.224 | -0.122 | -0.304 | 1.250 | -0.492 | -0.203 |
| PIP+TET+NEO | PIP+TET+ERY | PIP+TET+ERY_5 | -0.151 | -0.013 | 0.121 | -0.107 | -0.076 | -0.279 | -0.014 | 1.249 | -0.209 | -0.098 |
| PIP+TET+CHL | PIP+TET+ERY | PIP+TET+ERY_6 | -0.062 | 0.059 | 0.035 | -0.147 | -0.167 | -0.108 | 0.568 | 0.269 | -0.319 | -0.331 |
| PIP+TET+DOX | PIP+TET+ERY | PIP+TET+ERY_6 | -0.015 | -0.014 | 0.153 | 0.008 | -0.113 | -0.025 | -0.018 | 1.336 | 0.054 | -0.167 |
| PIP+TET+ERY | PIP+TET+ERY | PIP+TET+ERY_6 | -0.052 | -1.255 | 0.235 | -0.886 | -0.599 | -0.067 | -0.444 | 2.168 | -0.671 | -0.251 |
| PIP+TET+NEO | PIP+TET+ERY | PIP+TET+ERY_6 | -0.201 | -0.018 | -0.004 | -0.196 | 0.005 | -0.286 | -0.022 | -0.006 | -0.306 | 0.277 |
| PIP+TET+CHL | PIP+TET+ERY | PIP+TET+ERY_7 | 0.122 | 0.160 | 0.051 | -0.079 | -0.312 | 0.797 | 1.049 | 0.414 | -0.219 | -1.512 |
| PIP+TET+DOX | PIP+TET+ERY | PIP+TET+ERY_7 | 0.090 | 0.092 | -0.008 | 0.017 | -0.099 | 0.465 | 0.683 | -0.019 | 0.064 | -0.672 |
| PIP+TET+ERY | PIP+TET+ERY | PIP+TET+ERY_7 | 0.054 | -1.010 | 0.354 | -0.689 | -0.917 | 1.157 | -0.403 | 2.031 | -0.662 | -0.474 |
| PIP+TET+NEO | PIP+TET+ERY | PIP+TET+ERY_7 | -0.044 | 0.061 | 0.070 | -0.065 | -0.117 | -0.102 | 0.546 | 1.127 | -0.175 | -0.270 |
| PIP+TET+CHL | PIP+TET+ERY | PIP+TET+ERY_8 | -0.072 | 0.092 | -0.001 | -0.261 | -0.269 | -0.137 | 0.689 | -0.001 | -0.610 | -0.643 |
| PIP+TET+DOX | PIP+TET+ERY | PIP+TET+ERY_8 | -0.193 | 0.042 | -0.019 | -0.233 | -0.094 | -0.359 | 0.290 | -0.032 | -0.538 | -0.172 |
| PIP+TET+ERY | PIP+TET+ERY | PIP+TET+ERY_8 | -0.179 | -0.511 | 0.443 | -0.346 | -0.519 | -0.363 | -0.283 | 1.866 | -0.525 | -0.308 |
| PIP+TET+NEO | PIP+TET+ERY | PIP+TET+ERY_8 | -0.302 | 0.029 | 0.120 | -0.252 | -0.106 | -0.600 | 0.371 | 1.238 | -0.557 | -0.130 |
| PIP+TET+CHL | PIP+TET+NEO | PIP+TET+NEO_1 | 0.057 | -0.022 | -0.017 | -0.167 | -0.194 | 0.456 | -0.030 | -0.032 | -0.385 | -0.509 |
| PIP+TET+DOX | PIP+TET+NEO | PIP+TET+NEO_1 | 0.181 | -0.009 | 0.025 | 0.179 | 0.004 | 1.198 | -0.014 | 0.419 | 0.921 | 0.022 |
| PIP+TET+ERY | PIP+TET+NEO | PIP+TET+NEO_1 | -0.452 | -1.827 | -0.054 | -1.510 | 0.532 | -0.468 | -0.540 | -0.047 | -0.780 | 0.533 |
| PIP+TET+NEO | PIP+TET+NEO | PIP+TET+NEO_1 | 0.076 | 0.040 | 0.032 | 0.283 | 0.173 | 0.499 | 0.645 | 0.744 | 1.560 | 1.891 |
| PIP+TET+CHL | PIP+TET+NEO | PIP+TET+NEO_2 | -0.235 | -0.096 | -0.023 | -0.384 | -0.074 | -0.324 | -0.109 | -0.034 | -0.580 | -0.080 |
| PIP+TET+DOX | PIP+TET+NEO | PIP+TET+NEO_2 | -0.111 | -0.143 | -0.043 | -0.025 | 0.229 | -0.164 | -0.163 | -0.058 | -0.039 | 1.570 |
| PIP+TET+ERY | PIP+TET+NEO | PIP+TET+NEO_2 | -1.196 | -3.232 | -0.476 | -4.010 | 2.733 | -0.621 | -0.626 | -0.226 | -0.876 | 0.705 |
| PIP+TET+NEO | PIP+TET+NEO | PIP+TET+NEO_2 | -0.198 | -0.135 | -0.062 | 0.177 | 0.510 | -0.453 | -0.164 | -0.105 | 1.764 | 4.112 |
| PIP+TET+CHL | PIP+TET+NEO | PIP+TET+NEO_3 | -0.036 | 0.032 | 0.017 | -0.386 | -0.394 | -0.051 | 0.504 | 0.266 | -0.591 | -0.584 |
| PIP+TET+DOX | PIP+TET+NEO | PIP+TET+NEO_3 | 0.020 | 0.044 | 0.052 | 0.177 | 0.083 | 0.145 | 0.459 | 0.578 | 0.835 | 1.081 |
| PIP+TET+ERY | PIP+TET+NEO | PIP+TET+NEO_3 | -0.103 | -0.881 | 0.384 | -0.902 | -0.770 | -0.123 | -0.400 | 5.389 | -0.692 | -0.304 |
| PIP+TET+NEO | PIP+TET+NEO | PIP+TET+NEO_3 | 0.040 | 0.020 | 0.021 | 0.332 | 0.267 | 0.236 | 0.673 | 0.862 | 1.789 | 5.012 |
| PIP+TET+CHL | PIP+TET+NEO | PIP+TET+NEO_4 | 0.151 | 0.052 | 0.074 | -0.182 | -0.399 | 1.183 | 0.388 | 0.713 | -0.443 | -1.275 |
| PIP+TET+DOX | PIP+TET+NEO | PIP+TET+NEO_4 | 0.193 | 0.063 | 0.058 | 0.170 | -0.080 | 1.383 | 0.557 | 0.635 | 0.806 | -0.307 |
| PIP+TET+ERY | PIP+TET+NEO | PIP+TET+NEO_4 | -0.265 | -2.052 | -0.009 | -1.806 | -0.022 | -0.236 | -0.538 | -0.008 | -0.791 | -0.005 |
| PIP+TET+NEO | PIP+TET+NEO | PIP+TET+NEO_4 | 0.063 | -0.065 | 0.081 | 0.192 | 0.100 | 0.651 | -0.080 | 1.186 | 1.730 | 0.778 |
| PIP+TET+CHL | PIP+TET+NEO | PIP+TET+NEO_5 | -0.009 | 0.059 | 0.059 | -0.080 | -0.169 | -0.014 | 0.659 | 0.582 | -0.138 | -0.284 |
| PIP+TET+DOX | PIP+TET+NEO | PIP+TET+NEO_5 | -0.038 | -0.042 | 0.148 | 0.175 | 0.102 | -0.054 | -0.051 | 1.455 | 1.341 | 0.960 |
| PIP+TET+ERY | PIP+TET+NEO | PIP+TET+NEO_5 | -0.192 | -2.027 | 0.199 | -2.362 | -0.870 | -0.125 | -0.530 | 1.470 | -0.815 | -0.167 |
| PIP+TET+NEO | PIP+TET+NEO | PIP+TET+NEO_5 | -0.174 | -0.101 | 0.040 | 0.154 | 0.358 | -0.257 | -0.113 | 1.134 | 2.115 | 9.892 |
| PIP+TET+CHL | PIP+TET+NEO | PIP+TET+NEO_6 | 0.027 | 0.111 | 0.138 | -0.228 | -0.455 | 0.248 | 0.927 | 1.393 | -0.411 | -0.805 |
| PIP+TET+DOX | PIP+TET+NEO | PIP+TET+NEO_6 | 0.088 | 0.069 | 0.044 | 0.178 | 0.008 | 0.635 | 0.643 | 0.404 | 0.781 | 0.061 |
| PIP+TET+ERY | PIP+TET+NEO | PIP+TET+NEO_6 | 0.206 | -1.535 | 0.176 | -2.248 | -1.618 | 1.366 | -0.473 | 1.332 | -0.807 | -0.399 |
| PIP+TET+NEO | PIP+TET+NEO | PIP+TET+NEO_6 | 0.182 | 0.055 | 0.094 | 0.191 | -0.095 | 1.334 | 0.731 | 1.312 | 0.978 | -0.213 |
| PIP+TET+CHL | PIP+TET+NEO | PIP+TET+NEO_7 | 0.251 | 0.130 | 0.083 | -0.075 | -0.430 | 1.413 | 1.010 | 0.712 | -0.186 | -2.526 |
| PIP+TET+DOX | PIP+TET+NEO | PIP+TET+NEO_7 | 0.233 | 0.176 | 0.059 | 0.119 | -0.248 | 1.425 | 1.444 | 0.520 | 0.476 | -1.331 |
| PIP+TET+ERY | PIP+TET+NEO | PIP+TET+NEO_7 | -0.165 | -1.438 | -0.422 | -2.238 | 0.027 | -0.113 | -0.470 | -0.264 | -0.837 | 0.026 |
| PIP+TET+NEO | PIP+TET+NEO | PIP+TET+NEO_7 | 0.197 | 0.076 | 0.083 | 0.319 | 0.028 | 1.344 | 1.261 | 1.190 | 1.741 | 0.115 |
| PIP+TET+CHL | PIP+TET+NEO | PIP+TET+NEO_8 | 0.242 | 0.123 | 0.113 | 0.329 | -0.042 | 1.654 | 0.770 | 0.745 | 1.229 | -0.157 |
| PIP+TET+DOX | PIP+TET+NEO | PIP+TET+NEO_8 | 0.119 | 0.156 | 0.069 | 0.257 | -0.031 | 1.317 | 1.636 | 0.573 | 1.302 | -0.070 |
| PIP+TET+ERY | PIP+TET+NEO | PIP+TET+NEO_8 | 0.067 | -0.637 | -0.086 | -1.385 | -0.805 | 1.254 | -0.306 | -0.071 | -0.775 | -0.381 |
| PIP+TET+NEO | PIP+TET+NEO | PIP+TET+NEO_8 | -0.009 | 0.004 | -0.003 | 0.127 | 0.135 | -0.012 | 0.309 | -0.003 | 1.733 | 237.613 |
